# Interactions between the tumor and the blood systemic response of breast cancer patients

**DOI:** 10.1371/journal.pcbi.1005680

**Published:** 2017-09-28

**Authors:** Vanessa Dumeaux, Bjørn Fjukstad, Hans E. Fjosne, Jan-Ole Frantzen, Marit Muri Holmen, Enno Rodegerdts, Ellen Schlichting, Anne-Lise Børresen-Dale, Lars Ailo Bongo, Eiliv Lund, Michael Hallett

**Affiliations:** 1 Department of Biology, Concordia University, Montreal, QC, Canada; 2 School of Computer Science, McGill University, Montreal, QC, Canada; 3 Department of Computer Science, UiT the Arctic University of Norway, Tromsø, Norway; 4 Department of Surgery, St. Olavs University Hospital, Trondheim, Norway; 5 Faculty of Medicine, The Norwegian University of Technology and Science, Trondheim, Norway; 6 University Hospital of North-Norway, Narvik, Norway; 7 Department of Radiology and Nuclear Medicine, Oslo University Hospital, Oslo, Norway; 8 Nordland Central Hospital, Bodø, Norway; 9 Department of Cancer, Oslo University Hospital, Oslo, Norway; 10 Department of Cancer Genetics, Oslo University Hospital, Oslo, Norway; 11 Institute of Community Medicine, UiT the Arctic University of Norway, Tromsø, Norway; University of Cambridge, UNITED KINGDOM

## Abstract

Although systemic immunity is critical to the process of tumor rejection, cancer research has largely focused on immune cells in the tumor microenvironment. To understand molecular changes in the patient systemic response (SR) to the presence of BC, we profiled RNA in blood and matched tumor from 173 patients. We designed a system (MIxT, Matched Interactions Across Tissues) to systematically explore and link molecular processes expressed in each tissue. MIxT confirmed that processes active in the patient SR are especially relevant to BC immunogenicity. The nature of interactions across tissues (i.e. which biological processes are associated and their patterns of expression) varies highly with tumor subtype. For example, aspects of the immune SR are underexpressed proportionally to the level of expression of defined molecular processes specific to basal tumors. The catalog of subtype-specific interactions across tissues from BC patients provides promising new ways to tackle or monitor the disease by exploiting the patient SR.

## Author summary

We present a novel system (MIxT) to identify genes and pathways in the primary tumor that are tightly linked to genes and pathways in the patient systemic response (SR). These results suggest new ways to tackle and monitor the disease by looking outside the tumor and exploiting the patient SR.

## Introduction

Breast cancer (BC) research has largely focused on understanding the intrinsic properties of the primary tumor in order to therapeutically target key molecular components that drive progression within the tumor epithelial cells [1]. For example, tamoxifen and trastuzumab target the estrogen and human epidermal growth factor receptors (ER, HER2) whose expression levels in tumors define the traditional clinical subtypes of BC. The vast majority of BC-related genomic studies have focused on bulk tumor samples that are expected to be enriched for neoplastic epithelial cells [2]. These efforts have produced subtyping schemes that classify patients into groups based on the similarity of expression of diverse molecular markers and processes [3–9] and generated gene signatures that can predict patient prognosis and benefit from therapy [10–13].

Cancers however are much more than an autonomous mass of epithelial cells. They constitute multicellular systems capable of bidirectional interactions with neighboring non-malignant cells and extracellular components i.e. the tumor microenvironment [14–16]. Tumor-microenvironmental interactions are necessary for tumor progression and drug sensitivity [16, 17] and are becoming better understood [18–21]. In fact, several genomics studies of the BC microenvironment, including our efforts, show that the microenvironment reflects its tumor and harbors prognostic information [22–24]. However, we also recently established that the primary tumor and its microenvironment does not harbor accurate prognostic signals in approximately 20% of BC patients [9]. Specifically, these patients are consistently misclassified by all hallmarks of breast tumors defining tumor epithelial cells (such as proliferation and ER status) and their microenvironment (such as the infiltration of immune cells, angiogenesis and fibroblast activation).

The systemic response (SR) in cancer patients refers here to the perturbations that occur in peripheral blood cells, which include immune effector cells and circulate throughout the body. The fact that a tumor exerts systemic effects (via eg soluble or exosomal factors) may provide an explanation for the clinical observation that patients with one tumor have an increased risk of developing several independent tumors, and that removal of primary cancer improves the survival of patients with distant metastases at the time of diagnosis [25]. In addition, since ER positive (ER+) BC tends to recur as long as 10–15 years after surgical removal of the tumor, it is important to understand systemic factors governing late recurrence and therapeutic approaches that target beyond the tumor site. In fact, there is a rapidly increasing understanding of the various means a tumor employs to favor metastasis in distant organs [26, 27]. For example, an “instigating” BC can exploit the patient SR so that otherwise-indolent disseminated tumor cells become activated [27–32]. The SR has also been investigated in BC at time of diagnosis. Specifically, our recent comparison of blood profiles of BC patients and matched controls yielded a gene signature that reports the presence of BC [33]. This diagnostic signature is specific to BC (i.e. the test classifies women with carcinoma other than breast as negative), and the composition of genes and enriched pathways in the signature suggest that a cytostatic immune-related signal in the SR of patients is associated with the presence of a tumor. Finally, recent evidence demonstrates that engagement of systemic immunity is critical to the process of tumor rejection in genetically engineered mouse models [34].

This study is the first large-scale genomics effort to study the molecular relationships between patient SR and primary tumor. We generated and analyzed expression profiles from peripheral blood and matched tumor cells in 173 BC patients. First, our results highlight how the patient SR is especially relevant to BC immunogenicity. Second, we present a novel tool entitled Matched Interactions across Tissues (MIxT) that starts by identifying sets of genes tightly co-expressed across all patients in each tissue. Then, MIxT identifies which of these gene sets and pathways expressed in one tissue are associated with gene sets and pathways in the second tissue by determining if their expression patterns in tumor and in the patient SR are tightly correlated. We find that there are very few such associations when all BC are considered. However, we do identify biological processes with significant associations between tumor and patient SR when we stratify our analysis by BC subtype. That is, we identify molecular processes in the tumor that are tightly co-expressed with (different) molecular processes in the SR across patients of a specific subtype. In particular, we detail how several tumor-permissive signals are associated between the tumor and SR of basal BC patients.

## Results

### A population genomic resource of blood and matched tumor cells from BC patients

**Fig 1:**
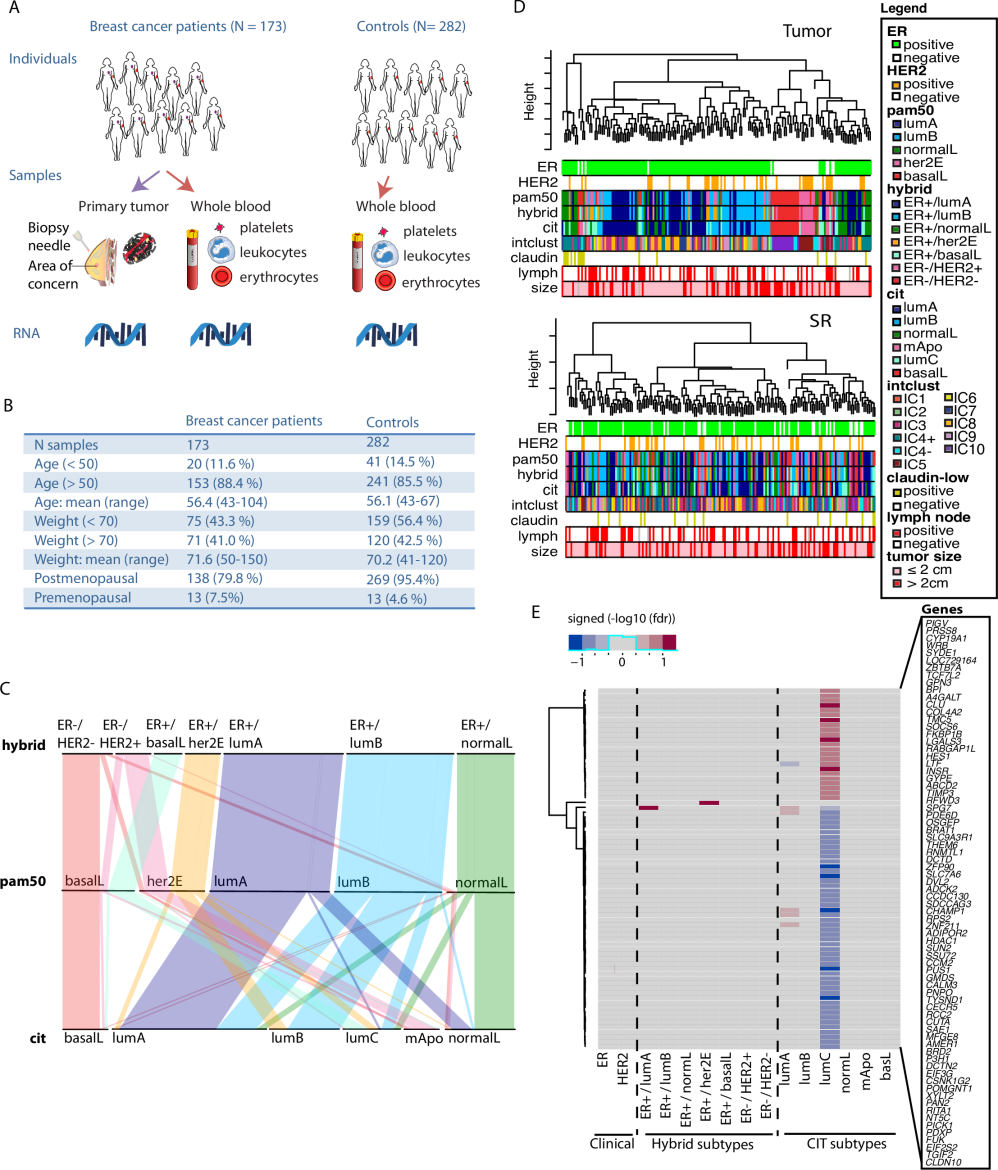
Individual characteristics and SR markers of BC subtypes. (A) Collection of biospecimen from BC patients and controls. (B) Individual characteristics of BC patients and controls. (C) Parallel plot displaying the repartition of BC patients across RNA-based subtyping schemes. (D) Sparse hierarchical clustering of BC patients based on genes expressed in tumor (upper) and the patient SR (lower). Clinicopathological and subtypes attributes are presented below the dendrogram. (E) Significant gene markers of subtypes in SR (false discovery rate, fdr *≤* 0.2). Blue and red shade correspond to under- and over- expression of the marker in a given subtype vs the others, respectively. Shading is proportional to the level of significance of the gene marker.

The Norwegian Women and Cancer (NOWAC) is a prospective population-based cohort that tracks 34% of all Norwegian women born between 1943–57. In collaboration with all major hospitals in Norway, we collected blood samples and matched tumor from women with an abnormal lesion, at the time of the diagnostic biopsy or at surgery, before surgery and any treatment (N ~ 300, S1 Text). RNA preservation for blood samples obtained followed our methodology previously described [33, 35] and detailed in S1 Text. RNA profiles from blood and tumor cells were generated using Illumina Beadarrays and data were processed following careful procedures (S1 Text, S1A Fig). After quality control, our study retained matched blood (SR) and tumor profiles of 173 BC patients diagnosed with invasive ductal carcinoma, and blood profiles of 282 control women (ie. women with no history of cancer with the exception of basal-cell and cervical carcinoma, which are both very common; Fig 1A). The controls are used to determine what constitutes a “normal” SR. BC patients and controls are comparable in terms of age, weight and menopausal status (Fig 1B). Several groups including ours have defined intra- and inter- individual variability of blood gene expression in healthy individuals [35–38]. All together, these studies demonstrate that intra-individual changes that can occur between blood draws are strikingly smaller than the variation observed among samples collected from different individuals. In this study, most women were 50 year-old or older and postmenopausal at time of sampling. Each profile measures the expression of 16,782 unique genes (S1 Text, S1A Fig). Almost all BC (95.4%) are early-stage disease (stage I or II).

### Transcriptional fingerprint of BC subtypes is not the predominant signal in the patient SR

Several tumor RNA-based subtyping tools were applied including PAM50 [5] that defines the intrinsic subtypes including luminal A (lumA), luminal B (lumB), normal-like (normalL), basal-like (basalL), and her2-enriched (her2E). The hybrid subtyping scheme partitions ER+ tumors according to their intrinsic subtype and partitions ER- tumors according to their HER2 status [9] (S1 Text, S1B and S1C Fig). In our dataset, all intrinsic luminals (lumA and lumB) and most normalL tumors (85.2%) are ER+; however, ~40% of basalL and ~50% of her2E BC are ER+ (Fig 1C, S1 Table). We also applied the Cartes d’Identité des Tumeurs (CIT) [8] subtyping scheme, which includes a ‘molecular-\ apocrine’ (mApo) subtype enriched for ER-/HER2+ tumors (78.6%) and the highly immunogenic ER+ luminal C (lumC) subtype enriched for ER+/basalL (39.1%). Fig 1C and S1 Table depict the relationships between these three schemes.

Although the IntClust (IC) subtyping scheme [6] is based on gene expression and DNA copy number profiles simultaneously, subtypes can be inferred using a reported RNA-based surrogate algorithm [7, 39]. S1 Table reports when subtypes from other schemes are enriched in each IC subtype. Most notably, IC1 and IC9 are enriched for CIT lumB; IC3, IC7 and IC8 are enriched for lumA; IC4+ is enriched for normalL and at lesser extent CIT lumC, IC5 enriched for mApo-her2E-HER2+, and IC10 enriched for basalL and ER-/HER2-. IC2, IC4-, and IC6 include very few patients (n < 10) and were therefore not further considered in our downstream analyses.

Restricting our attention to tumor profiles, we performed sparse hierarchical clustering with complete linkage using a permutation approach to select the tuning parameter that weights each gene to compute the dissimilarity matrix [40]. The resulting clusters were strongly associated with BC subtypes for all three RNA-based schemes (Fig 1D upper), which confirms that the transcriptional fingerprint of BC subtypes are also ubiquitous in our tumor samples. When restricting our attention to SR profiles, this unsupervised analysis does not identify patient clusters enriched for any given subtype across the three schemes (Fig 1D lower), suggesting that the transcriptional fingerprint of BC subtypes is not the predominant signal in the patient SR.

### Univariate gene markers are identified in the patient SR for one immunogenic BC subtype

We then asked if there are genes in the patient SR whose expression covaries with the state of the pathological variables ER and HER2 measured in the primary tumor. Although both are key drivers in BC, neither was found to be associated with individual gene expression changes in the patient SR (limma, linear models for microarray data, false discovery rate, fdr *≤* 0.2, Fig 1E; S1 Text)**.** Similarly, we asked if there are genes in the SR that are markers of tumor subtype (n patients > 10). For the intrinsic, hybrid, and IntClust subtypes, only the ubiquitin ligase *RFWD3* is highly expressed uniquely in the SR of lumA patients, and *TIMP3*, an inhibitor of matrix metalloproteinases, is highly expressed uniquely in ER+/her2E patients (Fig 1E, S2 Fig). For the CIT subtypes [8], we found 70 univariate gene markers in the SR of patients of the lumC subtype. The genes are primarily involved in general cellular processes such as protein processing or transcription in blood cells (fdr *≤* 0.2, Fig 1E, S3 Fig). The lumC subtype is defined by strong activation of several immune pathways at the site of ER+ tumor (i.e. antigen presentation and processing pathway, hematopoietic cell lineage, NK cell mediated cytotoxicity, T-cell receptor signaling and Toll-like receptor signaling) [8], suggesting that the SR is informative in cases where the primary tumor exhibits strong immune properties.

### Systems-level analysis reveals tissue-specific molecular processes

To compare genome-wide molecular changes in tumor and SR across patients, we used WGCNA-based clustering to define sets of tightly co-expressed genes (termed modules) in tumor and blood, respectively [41] (S1 Text). Briefly, we opted for a distance measure based on topological overlap, which considers the correlation between two genes and their respective correlations with neighboring genes [42] (S1 Text). The WGCNA cut and merge routine [43] after clustering identified 19 and 23 modules in the patient tumor and SR, respectively (S4 Fig; S1 Text). Each of these modules can be considered as a unique and stable pattern of expression shared by a significant number of genes.

Modules of the primary tumor are enriched for genes from a broad range of BC hallmarks including angiogenesis (salmon module), extracellular matrix reorganization (greenyellow), proliferation (green), and immune response (brown and darkturquoise) (S2 and S3 Tables, S1 Text). For example, the proliferation tumor module is enriched for mitotic cell cycle-related genes (green, n = 1064 genes; weight01 Fisher test [44], p-value < 2e-17) including the well-known marker of proliferation MKI67, 12 serine/threonine kinases that are used in the calculation of the mitotic kinase score (MKS) [45], and several components of the Minichromosome Maintenance Complex (MCM).

Modules of the patient SR are often enriched for genes involved in either general cellular processes such as translation (black) and transcription (grey60), or immune-related processes such as inflammatory response (brown, green), B-cell response (saddlebrown), innate immune response (greenyellow) (S4 and S5 Tables). Thus, seven SR modules are enriched in genes that are specifically expressed in immune cells [46] (“iris” signature set in S5 Table; Fisher’s Exact Test FET fdr < 0.05).

**Fig 2:**
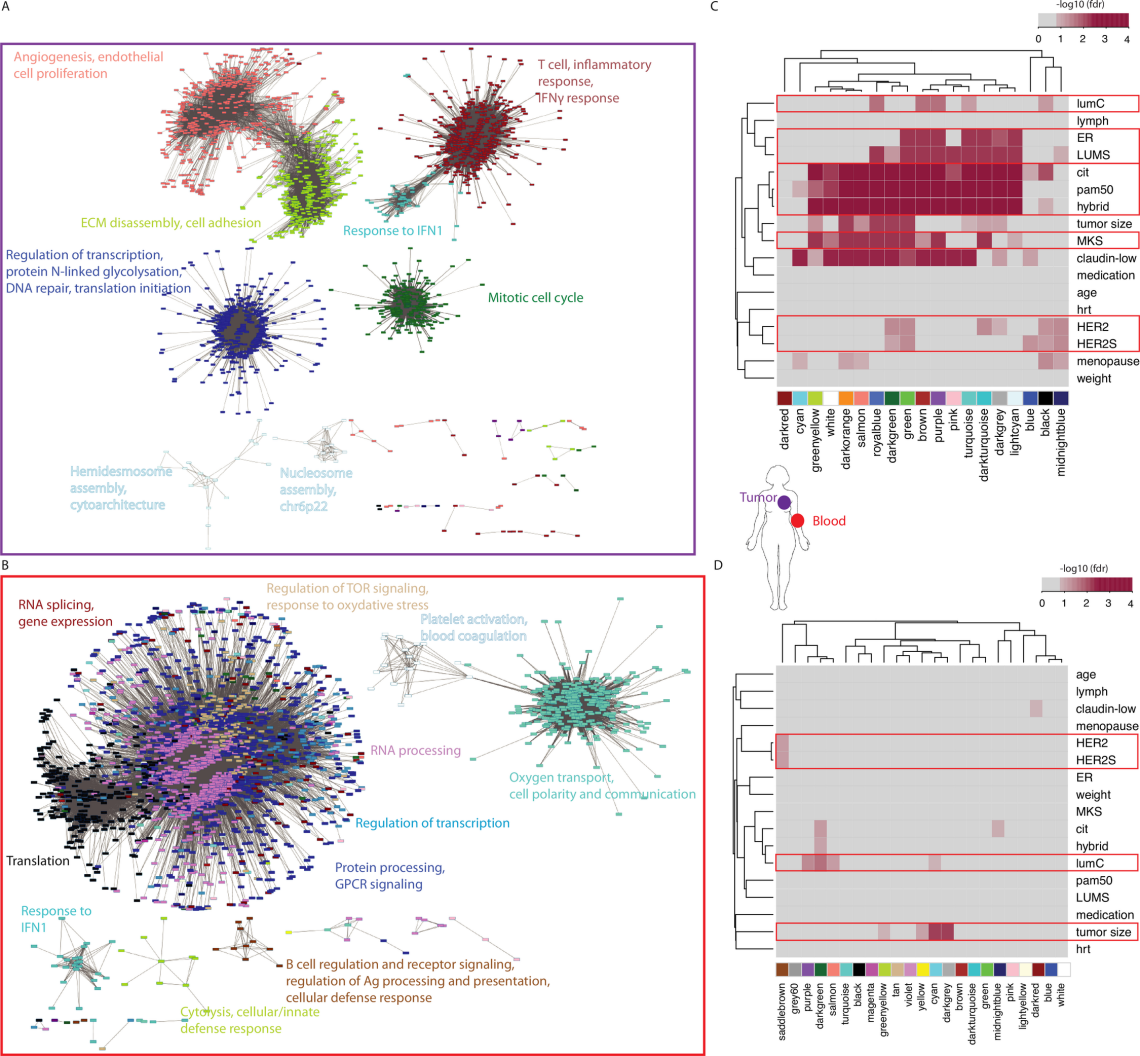
Gene co-expression networks, modules and associations with clinicopathological attributes of BC patients. (A) Network visualization using the edge-weighted spring embedded layout from Cytoscape (v3.2.1) including the top gene connections (topological overlap > 0.1) in tumor. Each node (gene) is color-coded by the module to which it belongs, Keywords representing top pathway enrichments (biological processes) are indicated for each module. (B) Network visualization including the top gene connections in the patient SR. The legend follows Fig 2A. (C) Associations between tumor modules and clinicopathological attributes of patients. Associations were estimated using Pearson correlation (Student’s p) or ANOVA. Shading is proportional to -log_10_(fdr) of the associations (fdr *≤* 0.15). HER2S: HER2 score; LUMS: luminal score; MKS: Mitotic kinase gene expression score; hrt: hormone replacement therapy (D) Associations between SR modules and clinicopathological attributes of patients. The legend follows Fig 2C.

We constructed a web-based system to visualize gene expression networks, heatmaps and pathway analyses of the modules in each tissue at http://mixt-blood-tumor.bci.mcgill.ca. In a network, genes are represented by nodes (colored by their module membership) that are connected by edges whose length corresponds to their level of co-expression across patients [47]. When selecting only strong gene-gene correlations (topological overlap > 0.1) and removing isolated nodes, the SR network has ~20% more genes than the tumor network (Fig 2A and 2B). Moreover, the SR network has approximately twice as many edges (89,465 connections between genes) than the tumor network (50,617 connections between genes). Thus, the underlying patterns of expression of the tumor genes (and modules) are more dissimilar from each other than the patterns of expression of the SR genes (and modules). In both tissues, the edges that span between modules reflect natural overlaps between cellular process (Fig 2A and 2B). For example in tumors, angiogenesis-related genes of the salmon module are strongly co-expressed with genes of the greenyellow module involved in extracellular matrix remodeling. In blood, modules enriched for genes involved in general cellular processes such as translation (black), RNA processing (violet), and RNA splicing (darkred) are also heavily connected to each other.

### Several processes in the SR are differentially expressed in patients with HER2+, lumC or large tumors

We first investigated the relationships between the expression pattern of each module and patient clinicopathological attributes. Towards this end, each gene of a module is used to rank the patient samples (S1 Text). In particular, the sum of gene ranks (ranksum) for each patient provides a linear ordering of the patient samples. Association tests then compare the ranksum values of patients with the attribute of interest eg tumor subtype (S1 Text).

When we consider tumor modules, the expression pattern of the green module (S5A Fig), previously established to be enriched for proliferation-related genes (S2 Table), ranks basalL, her2E and lumB tumors significantly higher than lumA and normalL tumors (ANOVA p-value < 1e-34, S5B Fig). In fact, we observe that the expression pattern of nearly every module is associated with BC subtype (15 of 19 modules, Fig 2C, fdr *≤* 0.15). Moreover, many tumor modules are associated with the proliferative state of the tumor encoded into the MKS score [45] (Pearson correlation, fdr *≤* 0.15) or with ER status (ER+ vs ER-, t-test, fdr *≤* 0.15), two variables that are strongly embedded in the definition of BC subtypes (Fig 2C). These results are consistent with our previous claim that patient subtype is a predominant signal in the primary tumor. Several tumor modules are associated with HER2 status of the tumor, however there are fewer such modules (n = 6) when compared with the proliferative state or ER status of the tumor (Fig 2C), suggesting that transcriptional fingerprint of HER2 is not as ubiquitous in tumor samples. A small number of modules are associated with the lumC subtype, including the brown module enriched for T-cell and inflammatory response genes (S2 Table). This is again consistent with the fact that this is a highly immunogenic subtype [8] (lumC versus not lumC, t-test, fdr *≤* 0.15, Fig 2C).

HER2 status, the lumC subtype and tumor size are all associated with modules of the patient SR (Fig 2D, t-test fdr *≤* 0.15). Although we did not find univariate gene markers in blood associated with HER2 status, the saddlebrown SR module is significantly underexpressed in patients with HER2+ tumors compared to other BC subtypes and controls (fdr = 0.07, S6A Fig) and is enriched for genes involved in B-cell receptor signaling and proliferation (including *BLK*, *CXCR5*, *CD19*, *CD79A*, *CD79B* and *FCRL5*; S4 and S5 Tables). Four SR modules are associated with the immunogenic lumC subtype; one of these modules are also associated with tumor size (Fig 2D, S6B and S6C Fig). Among the 70 univariate gene markers in blood of lumC tumors identified earlier, 31 are included in the darkgreen SR module predominantly underexpressed in lumC patients in comparison to other BC subtypes (fdr = 0.02, S6B Fig). In fact, all four SR modules associated with the lumC subtype are underexpressed compared to other BC subtypes and control samples (S6B and S6C Fig). This includes the purple module highly enriched for genes involved in T-cell (thymus) homing (*CCR7*, *LTA*, *LTB*, *VEGFB*, *HAPLN3*, *SLC7A6*, *SIRPG*, *BCL11B0*) and activation (*CD47*, *TNFRSF25*, *MAL*, *LDLRAP1*, *CD40LG*) which are underexpressed in lumC patients (fdr = 0.04, S6B Fig). Genes in the cyan modules are also found underexpressed in patients with large (> 2cm) tumors compared to other BC patients and controls (Fig 2D, S6C Fig). Finally, specifically for patients with large tumors, both the darkgrey module, which is enriched for *MYC* target genes, and the greenyellow module, which is enriched for genes involved in the lymphoid cell-mediated immunity (including *GZMH*, *GZMB*, *GZMM*, *KLRD1*, *PRF1*, *KLRG1*, and *GNLY*; S4 and S5 Tables), are underexpressed compared to the remaining BC patients and controls.

Together these results indicate that distinct SR are detected in BC patients with HER2+, lumC and/or large tumors, and that overall the patient immune response is underexpressed compared to patients of other subtypes and controls. These results also highlight the importance of distinct immune components for each of these disease groups. In particular, patients with HER2+ tumors exhibit low expression of genes specifically expressed in B-cell compared to patients with other BC subtypes. Patients with lumC tumors exhibit low expression of genes involved in T-cell homing and function compared to patients with other BC subtypes. Patients with large tumors (>2cm) exhibit low expression of genes involved in lymphoid cell-mediated immunity compared to patients with smaller tumors.

### Our Matched Interactions across Tissues (MIxT) approach explores biological processes that interact between tissues

Our analysis to this point identified modules within each tissue independently. Our focus here is on the relationships between tissues by asking if specific biologies in one tissue are correlated with (possibly distinct) biologies in the second tissue. To do this, we constructed a software entitled MIxT (Matched Interactions across Tissues) that contains the computational and statistical methods for identifying and exploring associations between modules across tissues (http://mixt-blood-tumor.bci.mcgill.ca).

**Fig 3:**
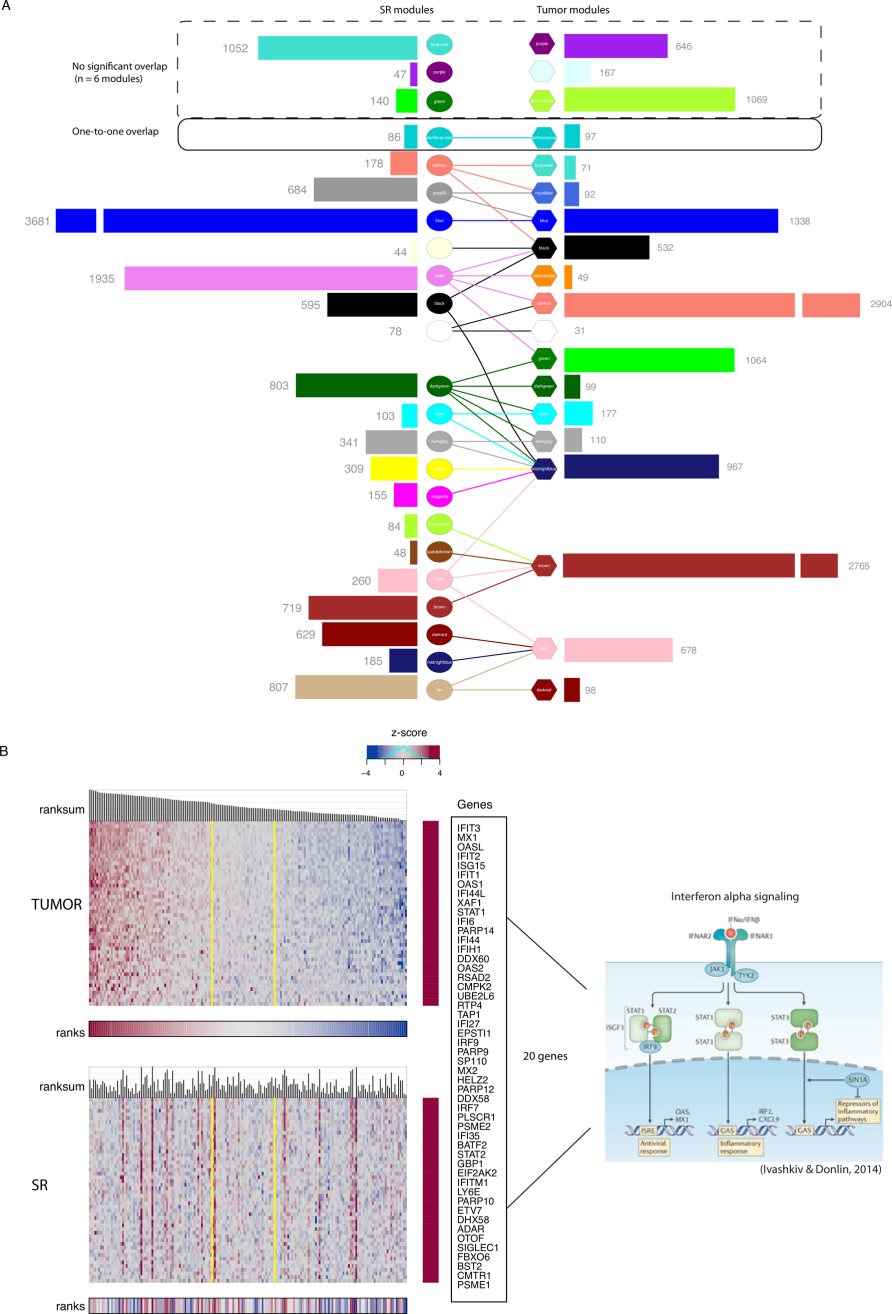
Modules size and overlap in their gene composition across tissues. (A) Histograms depicting number of genes composing modules in each tissue. Edges between modules indicate significant overlaps in gene composition (Fisher exact test, fdr < 0.01). (B) Expression heatmaps of the 47 genes included in both darkturquoise modules in tumor (upper) and SR (lower). Patients in both heatmaps are linearly ordered based on their ranksum of gene expression in tumors. Yellow vertical lines delimit the region of Independence (ROI_95_) in tumor that contains 95% of randomly generated samples. Twenty genes out of the 47 common genes are involved in the type 1 IFN signaling pathway (IFN alpha signaling pathway is depicted on the right).

Using MIxT, we first ask if genes that are tightly co-expressed in the primary tumor are also tightly co-expressed in the SR, and vice versa (Fig 3A, S1 Text) by investigating the gene overlap between tumor and SR modules (Fisher’s Exact Test FET, fdr < 0.01). Genes that retain strong co-expression across patients regardless of tissue type are likely to be involved in the same biological functions in both tissues as a “system-wide” response to the presence of the disease (even if patterns of gene expression across tissues might differ).

Most modules, regardless of tissue, have significant overlap with three to five modules in the other tissue (Fig 3A). In some cases, it appears that a single (large) module in one tissue is in large part the union of several smaller modules from the other tissue. For example, the brown tumor module has 2765 genes including many involved in immune-related processes (T-cell costimulation, the IFN-gamma pathway and inflammation, S2 and S3 Tables). All of these genes/processes show very strong co-expression in the tumor however, in the SR, these genes divide into four distinct patterns of co-expression (Fig 3A), captured by four different modules: brown (inflammation), greenyellow (cytolysis and innate immune response), saddlebrown (B-cell) and pink (TNFA inflammatory response) (S4 and S5 Tables).

Of note, MIxT identifies three modules in each tissue (SR and tumor) that do not have significant overlap with any module in the other tissue (Fig 3A). For tumors, this includes the purple module enriched for genes involved in estrogen response, the lightcyan module enriched for genes involved in hemidesmosome assembly and cytoarchitecture, and the greenyellow module enriched for genes involved in ECM organization (Fig 3A, S2 and S3 Tables). For the SR, this includes the turquoise module enriched for genes expressed in erythrocytes and involved in hemoglobin production, the purple module enriched for genes in translational termination, and the green module enriched for genes involved in inflammation and specifically expressed in myeloid cells (Fig 3A, S4 Table). This suggests that these processes and responses are either specific to a tissue type (eg ECM organization specific to tumor, and hemoglobin production specific to blood cells) or that the co-expression of genes involved in a defined process is unique to a particular tissue (eg genes specifically co-expressed in peripheral myeloid cells).

There is only one instance where a single tumor module has significant overlap with only a single SR module: darkturquoise modules of size = 86 and 97 genes in SR and tumor, respectively with 50 common genes, including 20 involved in the type 1 IFN signaling pathway (S2 and S4 Tables). Although these two “mirrored” modules share many genes, their patterns of expression are significantly different between the two matched tissues (Fig 3B, correlation between ranksums p-value > 0.05; S1 Text), hinting at a non-concordant expression of the local (in tumor) and systemic (in blood) IFN-1 mediated signals.

### MIxT identifies novel interactions between processes across tissues within specific subtypes

Whereas the previous section considers interactions defined by a large number of shared genes between a tumor and a SR module, we also examined more general notions of interactions in MIxT. Here we identify tumor and SR modules that have similar expression patterns (ie both modules linearly order the patients in very similar manner in both tissues) but do not necessarily share any genes in common. More specifically, MIxT derives estimates of significance for interactions using a random permutation approach based on the Pearson correlation between ranksums of gene expression in modules across tissues (S1 Text). This type of interaction detects a biological process or response in the primary tumor that is tightly correlated (or anti-correlated) with a (possibly distinct) biological process or response in the SR, and vice versa. The specific expression pattern in the tissues allows us to then postulate the functional nature of the interaction across tissues.

**Fig 4:**
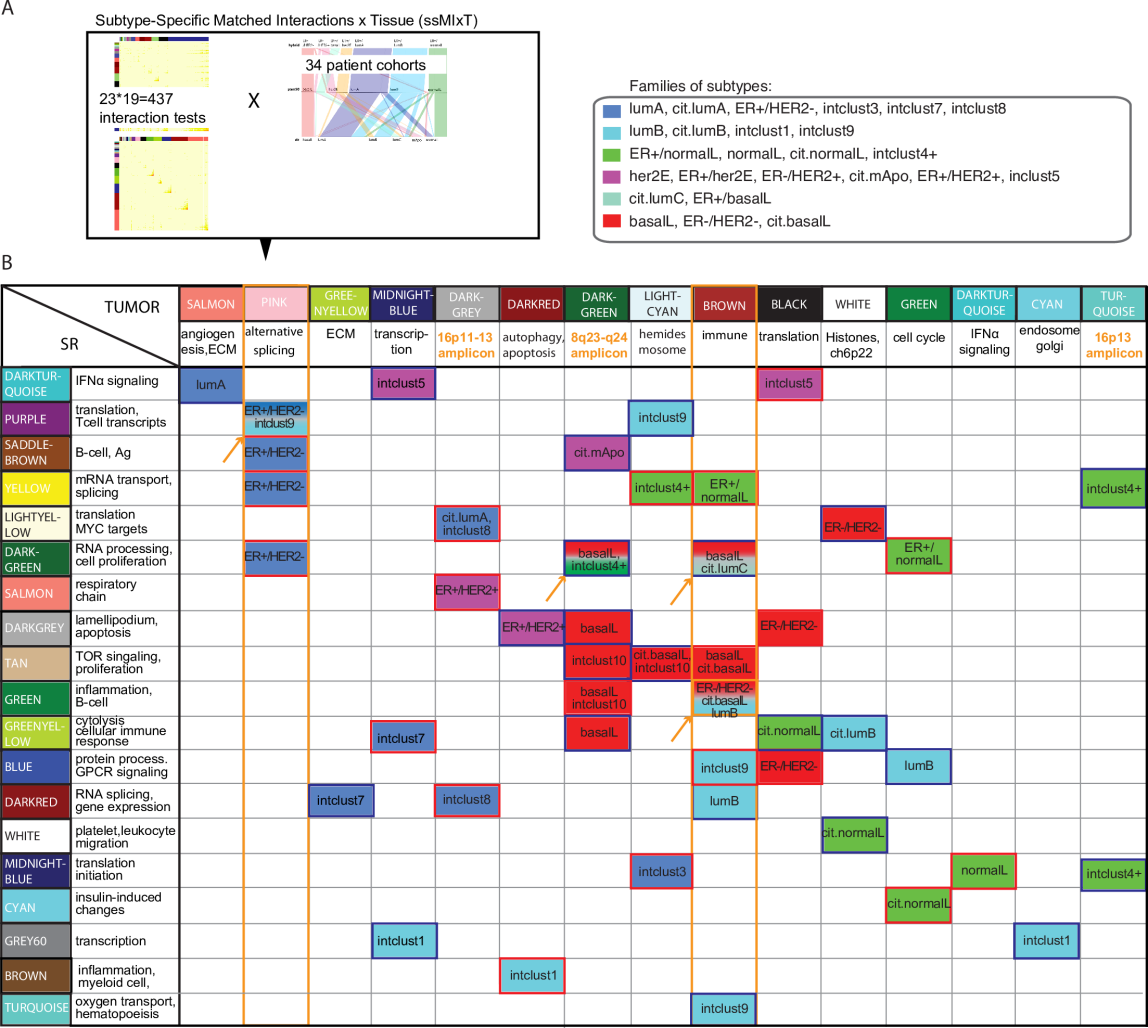
Subtype-Specific Matched Interactions across Tissue (ssMIxT). (A) Schematic of ssMIxT analysis (B) Significant associations between modules in SR and tumor from BC patients by subtype (MIxT statistic, p-value < 0.005). SR and tumor modules with top pathway enrichment keywords are presented in rows and columns, respectively. Subtype(s) in which the significant associations are found are indicated in the table. Blue and red borders correspond to negative and positive correlations between ranksums, respectively. Findings discussed in the text are highlighted in orange.

MIxT identified only one tumor module (of 19) that interacts with only a single SR module (of 23) across all patients (MIxT statistic; p-value < 0.005). The paucity of pan-BC interactions across tissues suggest the need to stratify by patient subtype. After stratification for each of the five subtyping schemes (clinical, PAM50, hybrid, CIT, and Intclust) (Fig 4A), we identified 53 interactions involving 15 tumor modules and 19 SR modules (MIxT statistic; p-value < 0.005; Fig 4B, S7 Fig). Tumor and SR modules are indicated in columns and rows of Fig 4B, respectively. A non-empty cell corresponds to a significant interaction with color used to indicate in which subtype the association is found, grouping together similar subtypes across schemes (eg basalL tumors of the pam50 and CIT schemes). Nearly all interactions are significant in only a single subtype (four exceptions indicated by orange arrows, Fig 4B). For some subtypes, a single stimulus in the tumor affects several biological processes in the patient SR. For example, within the ER+/HER2- subtype and only within this subtype, the pink tumor module, enriched for genes involved in alternative splicing, is associated with three SR modules, enriched for a diverse range of biological processes (orange rectangle in Fig 4B).

### Immune activity at the tumor site is associated with inflammatory SR in opposite ways for two distinct subtypes

The brown tumor module, which is enriched for genes involved in immune processes (S2 Table), has several interactions with SR modules across several subtypes (orange rectangle in Fig 4B). This includes interactions specific to normalL, lumB and IC9 but also several distinct interactions within the ER-/HER2- and basal subtypes. This suggests that immune signals expressed in tumor are associated with changes in expression of different molecular processes in the patient SR for a broad range of subtypes.

**Fig 5:**
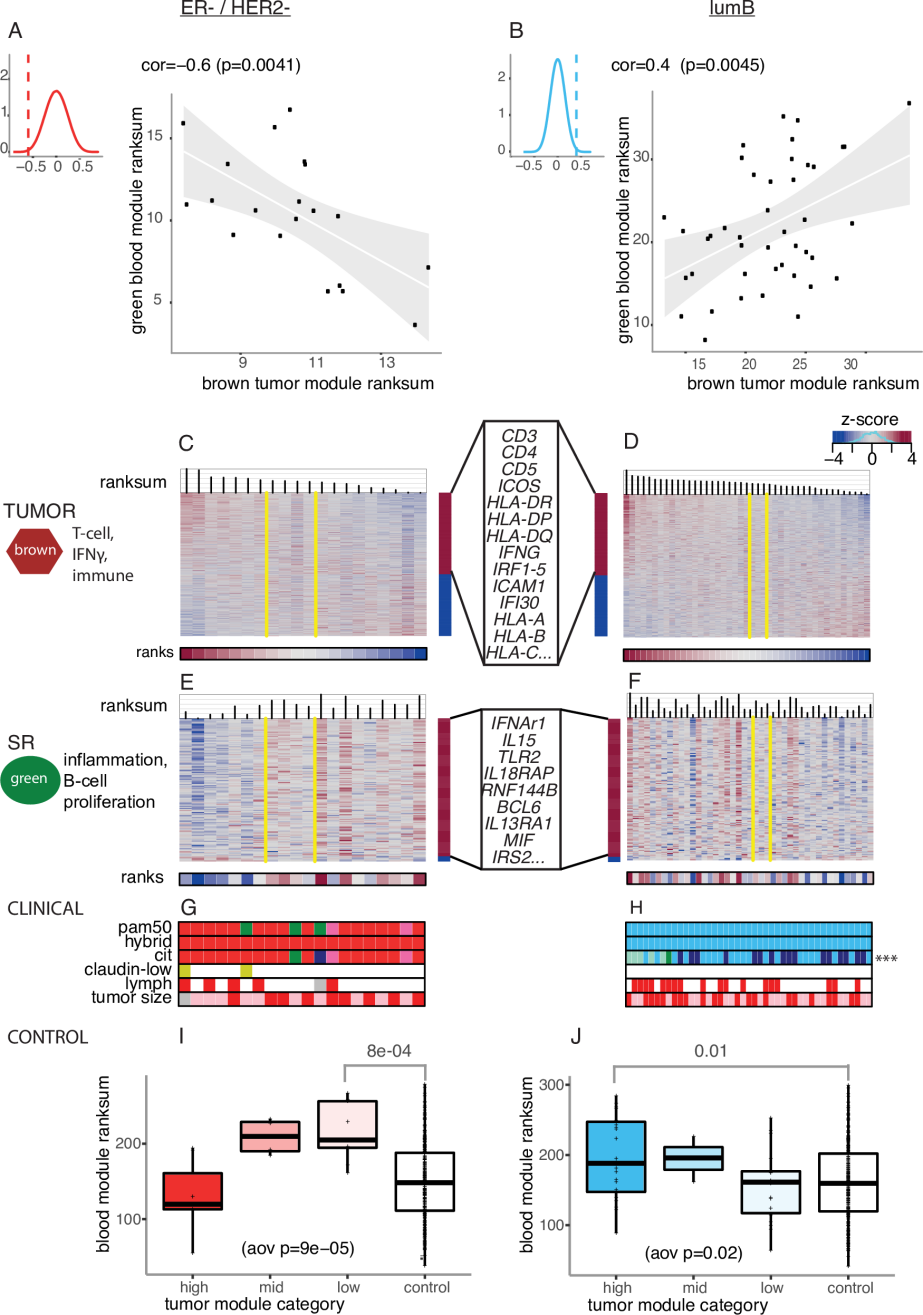
Association between the brown tumor and green SR module for two distinct subtypes. (A) Scatter plot of ranksums of the brown tumor module and the green SR module in ER-/HER2- patients. The top corner depicts the background distributions of the correlations coefficients between ranksums of every modules pairs across tissues in ER-/HER2- patients. (B) Scatter plot of ranksums of the brown tumor module and the green SR module in ER+/lumB patients. Legend follows Fig 5A (C) Expression heatmap of genes in the brown tumor module in ER-/HER2- patients. Patients are linearly ordered based on the ranksum of gene expression in the brown tumor module. Yellow vertical lines delimit the ROI_95_ in tumor that contains 95% of the randomly generated samples. Genes that are positively and negatively correlated with the ranksum are represented in the right sidebar colored in red and blue, respectively. Top pathway enrichment keywords and representative genes are indicated on the left and right of the heatmap, respectively). (D) Expression heatmap of genes in the brown tumor module in ER+/lumB patients. Legend follows Fig 5C. (E) Expression heatmap of genes in the green SR module in ER-/HER2- patients. Legend follows Fig 5C. Top pathway enrichment keywords and representative genes are indicated on the left and right of the heatmap, respectively. (F) Expression heatmap of genes in the green SR module in ER+/lumB patients. Legend follows Fig 5E. (G) Clinical characteristics of ER-/HER2- patients ordered by the ranksum of gene expression in the brown tumor module. Legend follows Fig 1D. (H) Clinical characteristics of ER+/lumB patients ordered by the ranksum of gene expression in the brown tumor module. Legend follows Fig 1D. Asterisks represent the level of significance of the associations between the gene ranksums for the brown tumor module and clinicopathological attributes of patients. Associations were estimated using ANOVA (fdr < ***0.01). (I) Distribution of ranksums for ER-/HER2- patients and controls induced by the expression of genes in the green SR module. Patients are grouped according to the ROI_95_ brown tumor module category as defined in Fig 5C. aov: analysis of variance (J) Distribution of ranksums for ER+/lumB patients and controls induced by the expression of genes in the green SR module. Patients are grouped according to the ROI_95_ brown tumor module category as defined in Fig 5D.

As alluded to earlier, only a few interactions are significant in two distinct subtypes simultaneously. For example, the brown tumor module is associated with green SR module in both ER-/HER2- and lumB although the directionality of the association differs between the two cases. More specifically, patients with high ranksums in the brown tumor module have low ranksums according to the green SR module, if the patient is of the ER-/HER2- subtype (Fig 5A, 5C and 5E, MIxT statistic, p-value = 0.004). At the same time, patients with high ranksums in the brown tumor module have high ranksum with respect to the green SR module, if the patient is of the lumB subtype (Fig 5B, 5D and 5F MIxT statistic, p-value < 0.004). In this manner the direction of correlation between the biological processes of the brown tumor module and of the green SR module is determined by the subtype of the patient.

For the brown tumor module in both subtypes, patients with a high ranksum (on the left of the ordering in Fig 5B or 5C for both subtypes) have the strongest immune signals in the tumors. This is because most of the immune-related genes in this brown module (within the red sidebar in Fig 5B and 5C, S3 Table) have highest expression in these patients. This includes genes involved in T-cell stimulation (incl. *CD3*, *CD4*, *CD5*, *ICOS*, several *HLA-DR*, *-DP*, -*DQ*), IFNɣ signaling (*IFNG*, *IRF1-5*, *ICAM1*, *IFI30*, *HLA-A -B -C*) and inflammation (incl. several interleukins, chemokines). For the green SR module in both subtypes, a high ranksum indicates an inflammatory SR (patients on the right in Fig 5E for ER-/HER2-, and patients on the left in Fig 5F for lumB). This is because almost every inflammation-related genes (incl. I*FNAR1*, *IL15*, *TLR2*, *IL18RAP*, *RNF144B)*, and B-cell proliferation genes (incl. *BCL6*, *IL13RA1*, *MIF*, *IRS2*) (within the red sidebar in Fig 5E and 5F, S5 Table) have highest expression in these patients.

Thus, ER-/HER2- patients with low immune activity at the tumor site have a high inflammatory SR (right side of Fig 5C and 5E). In fact, the level of the inflammatory response in these BC patients is higher than healthy controls (Fig 5I, t-test, p < 0.001). However, for the lumB subtype, the relationship between tumor and SR is reversed. Here, it is the patients that have high immune activity at the tumor site that have a high inflammatory SR (left side Fig 5D and 5F). In fact, the CIT subtyping scheme calls these patients on the left side as belonging to the lumC subtype (Fig 5H), the highly immunogenic ER+ subtype. In these lumB patients the inflammatory response is also higher than in healthy controls (t-test, p-value < 0.01; Fig 5J).

Altogether these results indicate that a high inflammatory SR is observed in both ER-/HER2- and ER+/lumB patients but increase in systemic inflammation is associated with distinct immune activity at the tumor site depending on subtype.

### Expression of genes in known BC amplicons is associated with concomitant changes in the patient SR for defined subtypes

Three tumor modules are enriched for genes within amplicons prevalent in BC [48] (highlighted in orange in Fig 4B, S3 Table). Two modules, the darkgrey and turquoise tumor modules, contain 68 genes (of 110) and 48 genes (on 71) located within the 16p11-13 amplicon highly prevalent in luminal tumors [48], respectively (S3 Table). The darkgrey module interacts with two distinct SR modules for the lumA and ER+/HER2+ subtype, respectively (S8A and S8B Fig). Tumors of both subtypes that over-express genes in the darkgrey module (left hand side S8C and S8D Fig) are likely amplified in 16p13. In these patients, the presence of this amplification is correlated with changes in expression of specific processes within the patient SR and these processes are distinct depending on subtype (S8E and S8F Fig, p < 0.005 in both cases). S8G and S8H Fig depicts associations between the presence of this amplification and patient clinico-pathological attributes. For example, in ER+/HER2+ patients (S8H Fig), the presence of 16p13 amplification is correlated with the luminal score of the tumor. In the lumA subtype, patients with the highest expression of the lightyellow SR module are significantly different than healthy controls (S8I Fig), and in the ER+/HER2+ subtype, patients with the lowest expression of the salmon module are significantly different than healthy controls (S8J Fig).

The third module enriched for genes involved in BC amplifications is the darkgreen tumor module. This module contains 43 (of 99) genes within the 8q23-24 amplicon prevalent in basal and her2E tumors [48] (S3 Table). Most associations with patient SR modules are specific to the basalL subtype (Fig 4B) and again suggest that basalL tumors that harbor this amplification have concomitant changes in expression of specific molecular processes in patient SR.

### A fully integrated view of molecular changes correlated between tumor and SR in basalL patients

**Fig 6:**
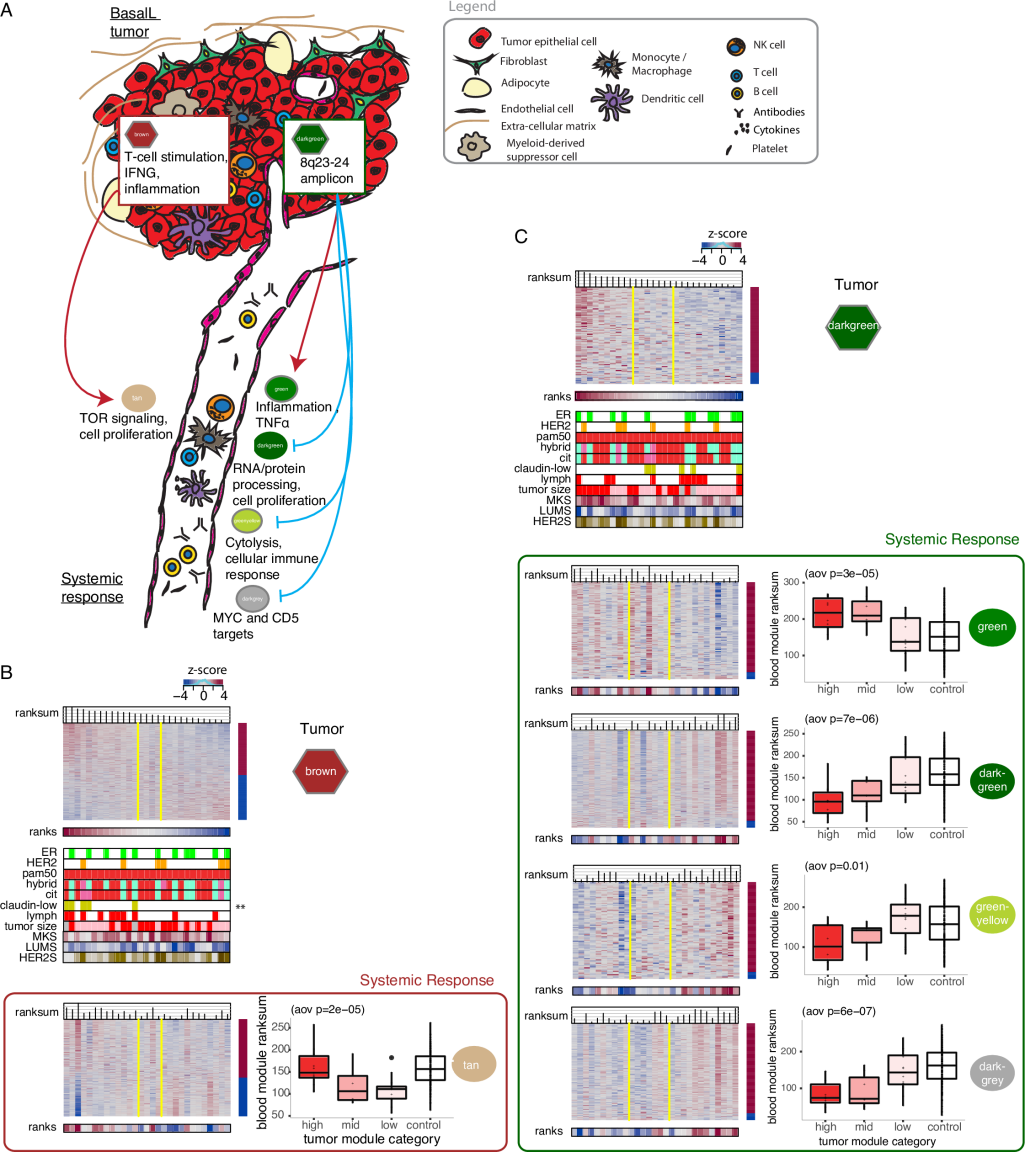
Significant Matched Interactions across Tissue (MIxT) in basalL patients. (A) The figure summarizes the two sets of significant MIxT in basalL patients detailed in Fig 6A and 6C. Top pathway enrichment keywords are presented for each module. Red and blue arrows correspond to negative and positive correlations between ranksums, respectively. (B) MIxT in basalL patients between the brown tumor module and the darkgreen SR module. Heatmaps are ordered by ranksum of gene expression in the brown tumor module. Asterisks represent the level of significance of the associations between the gene ranksums for the brown tumor module and clinicopathological attributes of patients (fdr < **0.05). Associations were estimated using ANOVA and Pearson correlation for categorical and continuous variable, respectively. Boxplots show the distribution of ranksums for the SR module in patients classified according to their ROI_95_ tumor module category and controls. (C) The second set of MIxT in basalL patients between the darkgreen tumor module and four SR modules (darkgreen, green, greenyellow, darkgrey). Legend follows Fig 6B.

Approximately one-fourth of the interactions identified by MIxT are specific to ER-/HER2-, IC10 and basalL subtypes, indicating that the tumor and SR interact strongly in this family of BCs (Fig 4B). We study two tumor modules in greater depth here: the brown immune-enriched module and the darkgreen 8q-enriched module, and their interactions with SR modules in basalL patients (Fig 6A–6C). Here the brown tumor module interacts with one (tan) SR module enriched for genes involved in TOR signaling and cell proliferation (Fig 6A and 6B). BasalL patients with low immune activity at their tumor site (right side of brown tumor module) have low expression of the tan SR module, and this expression is significantly lower than healthy controls (boxplots in Fig 6B, t-test p < 0.0005).

The darkgreen tumor module interacts with four SR modules in basalL patients (Fig 6A and 6C). High expression of genes in 8q is associated with high expression of the green SR module. This module is enriched for genes involved in inflammation. For the remaining three SR modules associated with the 8q-enriched tumor module, almost all genes in these modules are underexpressed when 8q genes are highly expressed (ie. the patient orderings are reversed compared to the darkgreen tumor module). These SR modules contain genes involved in general cellular processes of blood cells (RNA/protein processing, cell proliferation; darkgreen module), genes involved in cytolysis and lymphoid cell-mediated immunity (greenyellow module), and MYC and CD5 target genes (darkgrey module) (Fig 6A–6C, S5 Table). The increase in inflammatory SR and the decrease in the three other molecular processes in the SR of basalL patients whose tumor is amplified on 8q are all significantly different from how these processes are expressed in healthy controls (boxplots in Fig 6C). Overall, we identified one distinct signature in the SR of basalL patients with low immune activity at their tumor site and several immuno-suppressive signals in the SR of basalL patients whose tumor is amplified on 8q.

## Discussion

Molecular profiles of peripheral blood cells and matched tumors were generated and compared for a large cohort of BC patients part of the NOWAC study. The NOWAC consortium provides a highly curated population-based study with extensive gene expression profiling across several tissues from BC patients and controls [35, 49]. A careful design and our extensive experience in blood-based expression profiles enable a detailed molecular description of the patient SR to the presence of BC where blood molecular profiles represent effectively an “averaging” over the transcriptional programs of the different types of cells in blood.

We first asked if the SR could provide accurate univariate markers of tumoral properties such as ER status or subtype. Although thousands of transcripts are differentially expressed in tumors between ER+ and ER- BC [9, 50], there is no gene in SR that can reliably predict ER status of the primary tumor. Moreover, the SR does not inform on the intrinsic BC subtype of the tumor such as lumA, lumB or basalL subtype or on IntClust subtypes. Interestingly, univariate markers in the patient SR were only identified for the CIT lumC subtype defined as particularly immunogenic ER+ tumors [8], suggesting that the SR is informative in cases where the primary tumor exhibits strong immune properties. This is consistent with previous reports that uses blood transcriptomics as a gateway into the patient immune system [51–53] and which is extensively used in the context of autoimmune and infectious diseases [54–56]. This result suggests that it is also applicable in cancer such as BC.

To further investigate the molecular changes in the patient SR, we extended our analyses to multivariate approaches where genes are combined into sets or “modules”. In particular, we performed cluster analysis to partition the genes of both tumor and SR profiles into modules with each module representing a distinct pattern of expression across patients. Our user-friendly website (www.mixt-blood-tumor.bci.mcgill.ca) provides access to these modules built in each tissue, enables investigation of their expression profiles in each tissue and allow user-defined queries of gene, gene sets, and pathway of interest. Further, our MIxT approach estimates gene module expression in both tissues and find significant associations between modules across tissues in a representative cohort of BC patients.

In our dataset, the primary tumor and SR have approximately the same number of modules (19 and 23, respectively) but their gene composition is qualitatively different. Not surprisingly, many modules in tumors were enriched for genes involved in hallmarks of cancer, while SR modules were enriched for either general cellular processes or specific immune responses. Only one module involved in the IFN-I pathway is highly conserved in both tumor and SR, although the common genes had markedly different expression patterns between the two tissues. This is important as it establishes that genes, whose expression patterns may act as good markers in the primary tumor, are not necessarily expressed in the same manner within blood cells.

Our multivariate approach was able to identify modules from the patient SR that could reliably identify not only lumC but also HER2+ and large (> 2cm) tumors. These three cases are among the most immunogenic subtypes of BC and are of relatively poor prognosis. For these patients, gene expression in blood cells is mostly decreased compared to other BC and controls. This result also highlights the importance of distinct immune components of the SR for each of these disease groups: B-cells for HER2+ tumors, T-cells for lumC, and aspects of the cellular immune response for large tumors. Interestingly, a previous study showed that her2E tumors have the highest B-cell infiltration and expression of B-cell receptor gene segments, although this was not predictive of improved patient survival [57]. Our study finds an impaired systemic B-cell response specifically in HER2+ patients, consistent with an inefficient anti-tumoral response in these patients, potentially due to a dysfunctional antigen receptor response and cell development. We could also speculate that the dysfunctional thymic T-cell homing signature in lumC patients reflects the well-documented effect of estrogen on thymic T lymphopoiesis [58–61] in patients diagnosed with a highly immunogenic ER+ tumor. These associations would certainly require validation in follow-up studies.

Finally, MIxT focuses on molecular associations between tissues and provides a holistic view of molecular changes in BC patients. Although the focus here is towards gene expression of blood and matched tumor, our approach could be extended to multiple tissues (eg. blood-microenvironment-tumor) or other levels of molecular data (eg. DNA level somatic aberrations, gene and miRNA expression, epigenetic profiles).

Interestingly, associations between BC tumor and patient SR are heavily dependent on subtype. Only one interaction between tumor and patient SR is identified when all BC patients are considered in the analysis but many are identified when we first stratify patients by BC subtype. This is perhaps not surprising given that there is a great deal of molecular heterogeneity between BC subtypes making “one SR fitting all” highly unlikely. We identified molecular stimuli in tumors that change patient SR in multiple ways only for patients within a particular subtype. For example, expression of genes involved in alternative splicing in ER+/HER2- tumors is associated with changes in expression of multiple processes in SR of patients and those associations are observed only within this specific subtype.

Of note, immune signals measured at the tumor site are associated with distinct SR across a broad range of subtypes. Immune-related processes are known to be more or less expressed within every subtypes and have prognostic capacity in almost all subtypes [9]. Here we show that a change in immune activity at the tumor site is not associated with equal SR across subtypes. Furthermore, high immune signals in tumor is associated with the patient inflammatory SR in opposite ways depending if the patient is ER-/HER2- or lumB. The high inflammatory SR in ER-/HER2- patients (with low immune activity at the tumor site) and in lumB patients (with high immune activity at the tumor site) were both significantly different from how systemic inflammation is “normally” expressed in controls.

Finally, we identify other examples of interactions between tumor and patient SR that occur in subtype-specific fashions. In particular, three tumor modules were enriched for genes in known large-scale BC amplicons (16p11-13, 8q23-24). The expression of these genes changes in a coordinated manner from high to low, suggesting that these genes measure amplification of the corresponding region in BC tumors. In turn, these patterns of expression were associated with distinct SR depending on subtypes highlighting the significance of each amplicon in defining patient SR for particular BC subtypes (eg 16p13 in lumA and ER+/HER2+, and 8q23-24 in basalL and her2E). Of note, these patterns of expression also define patients with particular clinico-pathological characteristics. For example, ER+/HER2+ tumors that do not highly express the genes on 16p have a lower luminal score than ER+/HER2+ tumors that highly express the genes on 16p.

When we restrict our attention to basalL patients, we observe that both the immune-related module and the presence of a 8q23-24 amplification is associated with the patient SR. In fact, the subset of basal patients with 8q23-24 amplification exhibit high inflammatory SR and underexpress genes involved in general cellular proliferation of blood cells, in immune cytolysis, and in MYC and CD5 targets. Together, our matched profiles offer a detailed map of tumor-permissive SR particularly relevant for basalL tumors amplified on 8q and highlight a signature in the SR of basalL patients with low immune activity at their tumor site. This is especially interesting in the context of BC-immunotherapy combination or for monitoring response to these therapies. Overall, our study set the groundwork for further investigation of promising new ways to tackle and monitor the disease by looking outside the tumor and exploiting the patient SR.

## Materials and methods

### Gene expression data

Tumor and blood samples were obtained as part of the NOWAC study [49, 62] with approval from Regional Committees for Medical and Health Research Ethics in Norway. Between 2006–10, we collected blood and biopsy samples from BC cases at time of diagnosis, and blood samples from selected age-matched blood controls together with associated lifestyle and clinicopathologic data (S1 Text). In total, and after data preprocessing, profiles include 16,792 unique genes expressed in primary tumors and blood from 173 BC patients, and in blood from 290 controls (S1A Fig).

### Subtypes and gene markers of subtypes

We used ER status as measured by IHC and HER2 status measured by FISH or IHC where available. When unavailable, ER and HER2 status was determined using gene expression of the *ESR1* gene and 6 gene members of the HER2 amplicon, respectively [9, 63] (S1 Text, S1B and S1C Fig). In addition, we calculated the HER2 score (HER2S) and the luminal score (LUMS) as the average expression of the HER2 amplicon gene members and the pam50 luminal genes, respectively. A proliferation score was calculated similarly using 12 mitotic kinases to produce the Mitotic kinase gene expression score (MKS) [45]. Samples were labeled according to our subtyping schemes from the literature: PAM50 [5], hybrid [9], CIT [8], IntClust [7, 39] (S1 Text).

Lists of differentially expressed genes in SR according to subtypes were obtained using the R/Bioconductor package Limma [64]. Whenever p-values were adjusted for multiple testing, the false discovery rate [65] was controlled at the reported level (S1 Text).

### Weighted gene co-expression analysis (WGCNA) and gene modules

An unsigned weighted co-expression network was constructed independently in each tissue (SR and tumor) using the R/Bioconductor package WGCNA [41] (S1 Text). First, a matrix of pairwise correlations between all pairs of genes is constructed across blood and tumor samples, respectively. Next, the adjacency matrix is obtained by raising the co-expression measure to the power β = 6 (default value). Based on the resulting adjacency matrix, we calculate the topological overlap, which is a robust and biologically meaningful measure of network interconnectedness [42] (that is, the strength of two genes’ co-expression relationship with respect to all other genes in the network). Genes with highly similar co-expression relationships are grouped together by performing average linkage hierarchical clustering on the topological overlap. The Dynamic Hybrid Tree Cut algorithm [43] cuts the hierarchal clustering tree, and modules are defined as branches from the tree cutting. Modules in each network were annotated based on Gene Ontology biological processes (weight01 Fisher test [44]), MSigDB [66] and other curated signatures relevant to immune and blood cell responses [33, 46, 52] (S1 Text)

### Gene ranksum and linear ordering of patients

Our approach maps samples to a linear ordering based on expression of genes within a given module or signature of interest (S1 Text). In an univariate fashion, each gene within a given module/signature is used to rank all patients based on their expression. For each patient, the ranks of all k genes from the signature are summed and patients are then linearly ordered from right to left according to this ranksum vector. To identify the left and right boundaries of the low and high regions within the observed linear ordering, we delimit the region of independance (ROI_95_) for each module. Briefly, we compute (n = 10K times) the position of an artificial patient within the observed linear ordering by summing the randomized ranks over all k genes in the module (S1 Text). The ROI_95_ is defined as the region that contains 95% of the randomly generated samples. The three defined categories of patients correspond to those patients that have high ranskums of the module/signature (high category), low ranksums of the module/signature (low category), and a set of patients where the expression of the genes within the module/signature lose their pattern of pairwise correlation (mid category).

### Module association tests

Using gene ranksums to capture module expression, we asked how modules are associated with patients’ clinical attributes and how they are associated across tissues. Pearson correlation and Analysis of Variance (ANOVA) was used to test association between a given module and continuous patient attributes (eg. age, weight, MKS, LUMS) and between a given module and categorical patient attributes (eg. ER, HER2, subtypes, lymph node status), respectively (S1 Text). For each variable. we computed empirical p-values after permuting clinical labels 1000 times. For each variable, we perform a total of 42 association tests (23 blood modules + 19 tumor modules) and used false discovery rate [65] to correct for multiple testing for each variable independently or for each “family” of tests when dependent variables are very similar (S1 Text).

Interactions between modules across tissues are identified using a random permutation approach based on the Pearson correlation between ranksums of gene expression in modules across tissues (S1 Text). ANOVA was used to compare SR module expression between BC patients (assigned to a given tumor module ROI_95_ categories) and controls.

### Data and software availability

#### Data resource

Microarray data have been deposited at the European Genome-phenome Archive [67] (EGA; https://www.ebi.ac.uk/ega/; accession number EGAS00001001804).

#### Software

The MIxT web application (http://mixt-blood-tumor.bci.mcgill.ca/) is written in the Go programming language to provide an interface to statistical analyses in R and link to online databases. Users can browse through all the results generated for this study, visualize gene co-expression networks and expression heatmaps, and search for genes, gene lists, and pathways. We use Bootstrap (http://getbootstrap.com) to build the user interface and Javascript libraries D3 (http://d3js.org) and Sigma (http://sigmajs.org) to build interactive visualizations. The web application framework is open sourced at http://github.com/fjukstad/mixt.

## Supporting information

S1 Table Enrichment of clinicopathological and tumor subtypes attributes across subtyping schemes. The table shows statistically significant associations between tumor attributes (columns) and subtypes (rows). For columns representing binary variables (ER, HER2, LN, as well as subtype/cohorts), the table shows the number of patients and the level of significance computed using Fisher's exact test (FET). Enrichment is indicated using “+” symbols, while for depletion “-” symbols are used. The number of symbols in each entry correspond to significance levels of 0.05, 0.01, 0.001, and < 0.0001. For example, the entry in row “her2E” and column “HER2+” contains the symbol “++++” indicating that herE patients are more likely to be HER2+ than non-her2E patients. In contrast, the entry in row “her2E” and column “ER+” contains the symbol “—” indicating that her2E patients are less likely to be ER+ than non-her2E patients. Grey indicates cases where enrichment cannot be calculated.
(XLSX)

S2 Table Top GO terms enriched in tumor modules. Top 5 GO terms that overlap with each module. “Annotated” indicates the number of genes in the GO term, “Significant” indicates the number of overlapping genes. “Expected” indicates the number of genes that we would expect by chance to be overlapping with the GO term. “classicFisher” presents the p-value from a classic fisher exact test and “weight01Fisher” presents the p-value from the weight01 algorithm and fisher exact test from [44].
(XLSX)

S3 Table Top 5 enrichments among each of the following signature sets. i) c1, c2.cgp, c2.cp, c6, c7 and h gene set collections from MSigDB signatures (v5.1) [66]. ii) peripheral-blood mononuclear cell (PBMC) transcriptional modules (sig.set = i) from [52]. iii) our blood-based gene expression signatures (341- and 50-gene; sig.set = d) for BC [33] iv) immune-specific gene sets (sig.set = iris) from [46]. Enrichment for each gene signature was estimated for all genes in the modules and for genes that are positively (red genes up) or negatively (blue genes dn) correlated with the patient ranksum only using the hypergeometric minimum-likelihood p-values, computed with the function ‘dhyper’ (equivalent to one-sided Fisher exact test). P-values were then adjusted for multiple testing using false discovery rate [65].
(XLSX)

S4 Table Top GO terms enriched in SR modules. Legend follows S2 Table.
(XLSX)

S5 Table Top 5 gene sets of each signature set enriched in SR modules. Legend follows S3 Table.
(XLSX)

S1 Fig Gene expression and clinical data processing. (A) Preprocessing of the microarray data was performed identically in each of the five datasets: blood (bl) 1–4 and tumor (t. 1) datasets. Steps that trim samples and probes/genes are presented horizontally and vertically, respectively. In total, we investigated blood and tumor profiles from 173 BC patients and blood profiles from 282 controls. Profiles include 16,782 unique genes. (B) Imputation of missing ER status based on expression of *ESR1* gene. Receiver operating characteristic (ROC) curve setting on the right using IHC/FISH assignment as true label. False positive rate threshold was set to < 0.2 with regard to the true label. (C) Imputation of missing HER2 status based on expression of genes included in the HER2 amplicon (*ERBB2*, *GRB7*, *PGAP3*, *PNMT*, *MIEN1*, *TCAP*).
(TIF)

S2 Fig Significant univariate gene markers of subtypes in SR (false discovery rate, fdr *≤* 0.2). Blue and red shade correspond to under- and over- expression of the marker in a given subtype vs the others, respectively. Shading is proportional to the level of significance of the gene marker.
(TIF)

S3 Fig Gene expression heatmap of the 70 blood markers of lumC tumors. Rows correspond to genes and columns correspond to samples. Gene expression are scaled by row. Patients are linearly ordered based on their ranksum of gene expression. Genes are ordered by their correlation to the observed patient ordering. Genes that are positively and negatively correlated with the patient ranksum are represented in the right sidebar colored in red and blue, respectively. Yellow vertical lines delimit the Region Of Independence (ROI_95_) that contains 95% of the randomly generated samples. A green tick in ‘lumC’ refers to a patient with a luminal C tumor according to the CIT scheme [8].
(TIF)

S4 Fig Gene co-expression networks in each tissue. (A) Heatmap of the topological overlap between genes expressed in tumors. Each row and column represent a gene, light color indicates low topological overlap and progressively darker red indicates higher topological overlap. Module assignment is displayed along the left and the top of the heatmap. (B) Heatmap of the topological overlap between genes expressed in SR. The legend follows S4A Fig.
(TIF)

S5 Fig Expression patterns of the green tumor module. (A) Expression heatmap of genes in the green tumor module. Legend follows S3 Fig. Color coding for ER, HER2, pam50, hybrid, cit, claudin-low and lymph follows Fig 1D. In general, a tick for a binary clinical variable refers to a positive value (eg. a red tick in ‘basalL’ refers to patients with basalL tumors). For continuous variables such as Mitosis Kinase Score (MKS), Luminal Score (LUMS), HER2 score (HER2S), age, and weight, dark and light shades represent high and low values, respectively. Asterisks represent the level of significance of the associations between the gene ranksums for the green tumor module and clinicopathological attributes of patients. Associations were estimated using ANOVA or Pearson correlation for categorical and continuous variable, respectively (p-value < *0.05, **0.01, ***0.001). (B) Distribution of ranksums for the green tumor module according to pam50 subtypes. aov: analysis of variance.
(TIF)

S6 Fig SR modules associated with clinico-pathological variable. (A) One (saddlebrown) modules in the patient SR is associated with HER2+ BC. (B) Three modules in the patient SR are associated with lumC BC. (C) One module in the patient SR are associated to both lumC and large (>2cm) tumors. (D) Three modules in the patient SR are associated with large (>2cm) tumors. The legend for expression heatmaps (left) follows S3 Fig. Boxplots (right) compare module expression in SR from patients with lumC, HER2+, or large tumors with other BC patients, and controls. aov: analysis of variance.
(TIF)

S7 Fig Background distributions of the correlations coefficients between ranksums of gene expression in modules across tissues within each subtype. The dotted lines represent the lower and higher bounds that were used to call significant associations between modules across tissues. Curves are colored according to the families of subtypes as listed in Fig 4.
(TIF)

S8 Fig Associations between the darkgrey tumor module and distinct SR by subtypes. (A) Scatter plot of ranksums of the darkgrey tumor module and the lightyellow SR module in CIT lumA patients. The top corner depicts the background distributions of the correlations coefficients between ranksums of every modules pairs across tissues in CIT lumA patients. (B) Scatter plot of ranksums of the darkgrey tumor module and the salmon SR module in ER+HER2+ patients. Legend follows S8A Fig (C) Expression heatmap of genes in the darkgrey tumor module in luminal A patients under the CIT scheme [8]. Patients are linearly ordered based on the ranksum of gene expression of the darkgrey tumor module. Yellow vertical lines delimit the ROI_95_ in tumor that contains 95% of the randomly generated samples. Genes that are positively and negatively correlated with the patient ranksum are represented in the right sidebar colored in red and blue, respectively. Top enrichment keywords are indicated on the left of the heatmap (S2 and S3 Tables). (D) Expression heatmap of genes in the darkgrey tumor module in ER+/HER2+ patients. Legend follows S8C Fig. (E) Expression heatmap of genes in the lightyellow SR module luminal A patients under the CIT scheme [8]. Legend follows S8C Fig. Top enrichment keywords are indicated on the left of the heatmap (S4 and S5 Tables). (F) Expression heatmap of genes in the salmon SR module in ER+/HER2+ patients. Legend follows S8C Fig. Top enrichment keywords are indicated on the left of the heatmap (S4 and S5 Tables). (G) Clinical characteristics of luminal A patients under the CIT scheme [8] ordered by gene ranksums derived from the darkgrey tumor module. Legend follows Fig 1D. Asterisks represent the level of significance of the associations between the gene ranksums for the darkgrey tumor module and clinicopathological attributes of patients. Associations were estimated using ANOVA (fdr < *0.1, **0.05, ***0.01). (H) Clinical characteristics of ER+/HER2+ patients ordered by gene ranksums derived from the darkgrey tumor module. Legend follows S8G Fig. (I) Distribution of ranksums for luminal A patients under the CIT scheme [8] and controls induced by the expression of genes in the lightyellow SR module. Patients are grouped according to the ROI_95_ darkgrey tumor module category as defined in S8C Fig. aov: analysis of variance (H) Distribution of ranksums for ER+HER2+ patients and controls induced by the expression of genes in the salmon SR module. Patients are grouped according to the ROI_95_ darkgrey tumor module category as defined in S8D Fig.
(TIF)

S1 Text Supporting methods. (PDF)
