# Supplementary figures and images for: Interactions between the tumor and the blood systemic response of breast cancer patients
Source: PLoS Comput Biol. 2017 Sep 28;13(9):e1005680. doi: 10.1371/journal.pcbi.1005680 (PMC5619688; doi:10.1371/journal.pcbi.1005680)

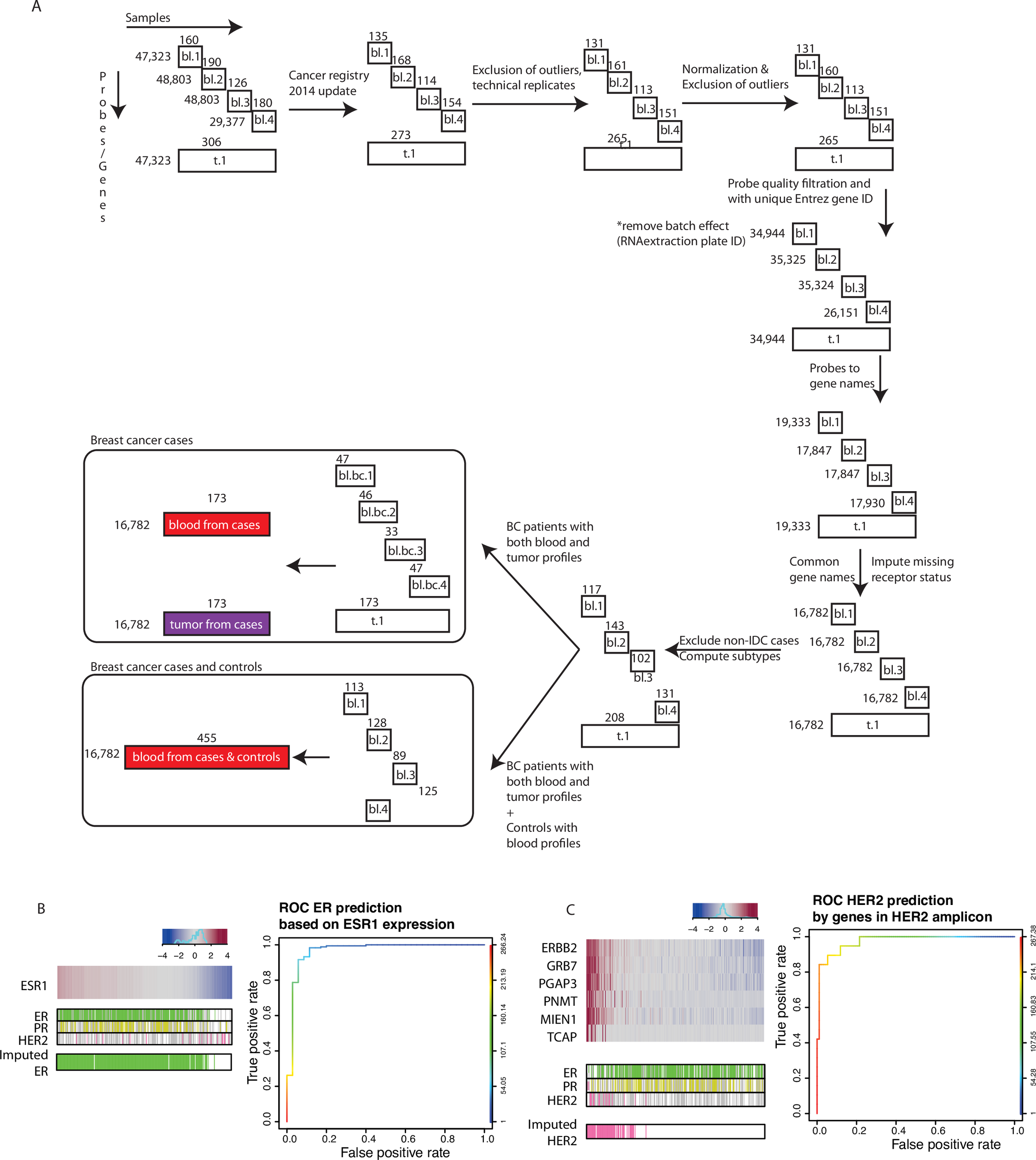

Supplement: S1 Fig — (A) Preprocessing of the microarray data was performed identically in each of the five datasets: blood (bl) 1–4 and tumor (t. 1) datasets. Steps that trim samples and probes/genes are presented horizontally and vertically, respectively. In total, we investigated blood and tumor profiles from 173 BC patients and blood profiles from 282 controls. Profiles include 16,782 unique genes. (B) Imputation of missing ER status based on expression of ESR1 gene. Receiver operating characteristic (ROC) curve setting on the right using IHC/FISH assignment as true label. False positive rate threshold was set to < 0.2 with regard to the true label. (C) Imputation of missing HER2 status based on expression of genes included in the HER2 amplicon (ERBB2, GRB7, PGAP3, PNMT, MIEN1, TCAP). (TIF) [file pcbi.1005680.s006.tif]

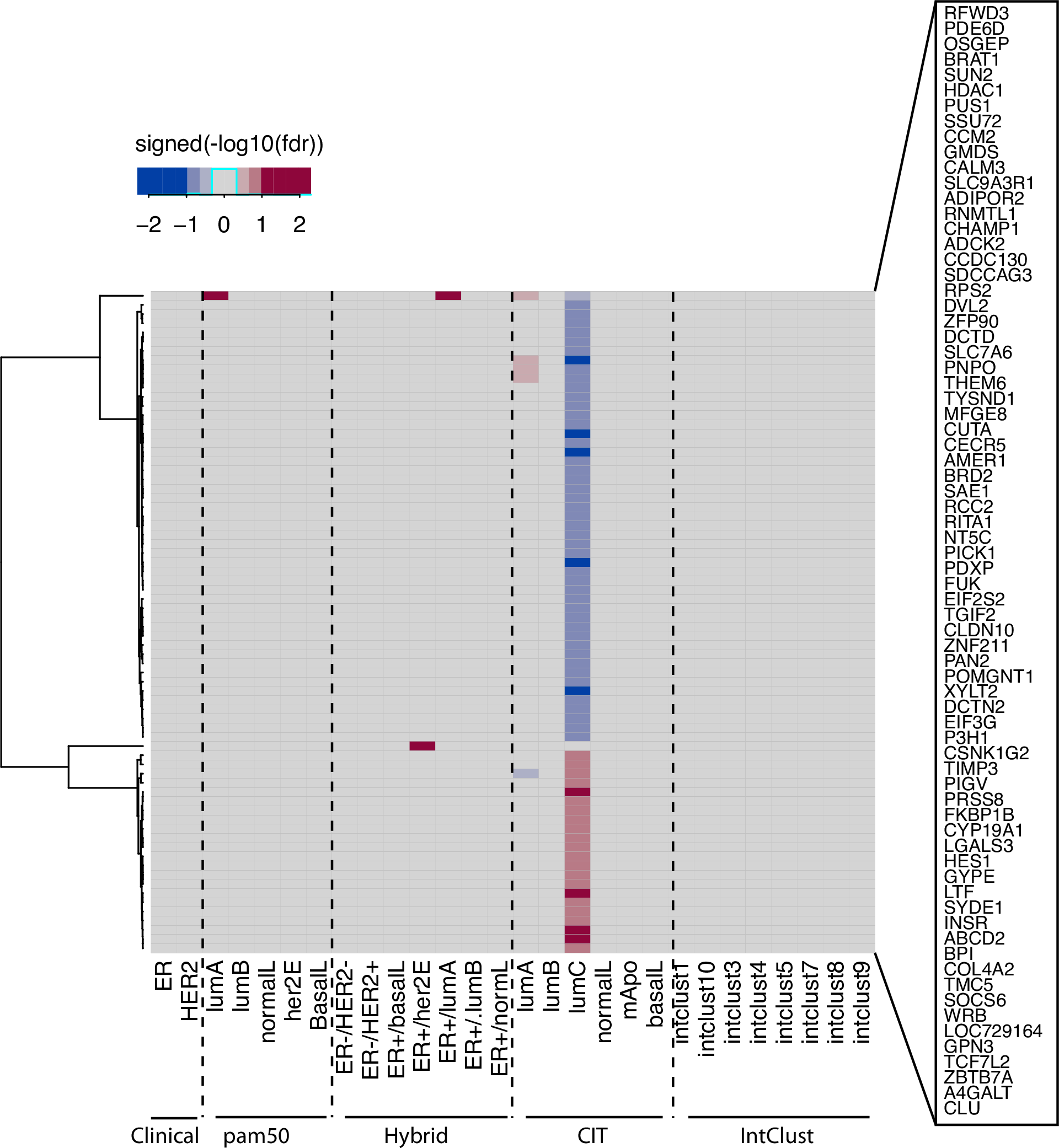

Supplement: S2 Fig — Blue and red shade correspond to under- and over- expression of the marker in a given subtype vs the others, respectively. Shading is proportional to the level of significance of the gene marker. (TIF) [file pcbi.1005680.s007.tif]

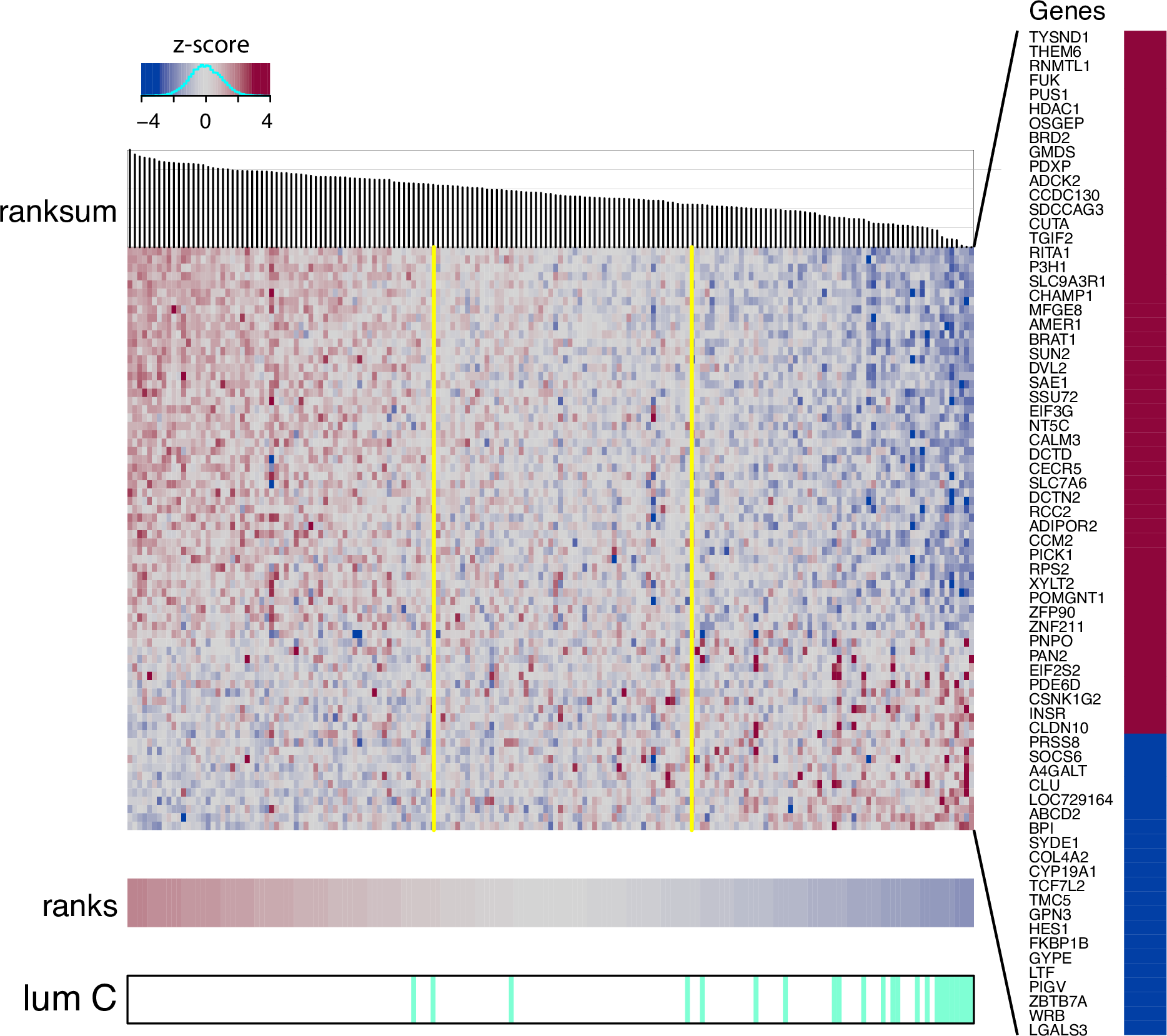

Supplement: S3 Fig — Rows correspond to genes and columns correspond to samples. Gene expression are scaled by row. Patients are linearly ordered based on their ranksum of gene expression. Genes are ordered by their correlation to the observed patient ordering. Genes that are positively and negatively correlated with the patient ranksum are represented in the right sidebar colored in red and blue, respectively. Yellow vertical lines delimit the Region Of Independence (ROI95) that contains 95% of the randomly generated samples. A green tick in ‘lumC’ refers to a patient with a luminal C tumor according to the CIT scheme [8]. (TIF) [file pcbi.1005680.s008.tif]

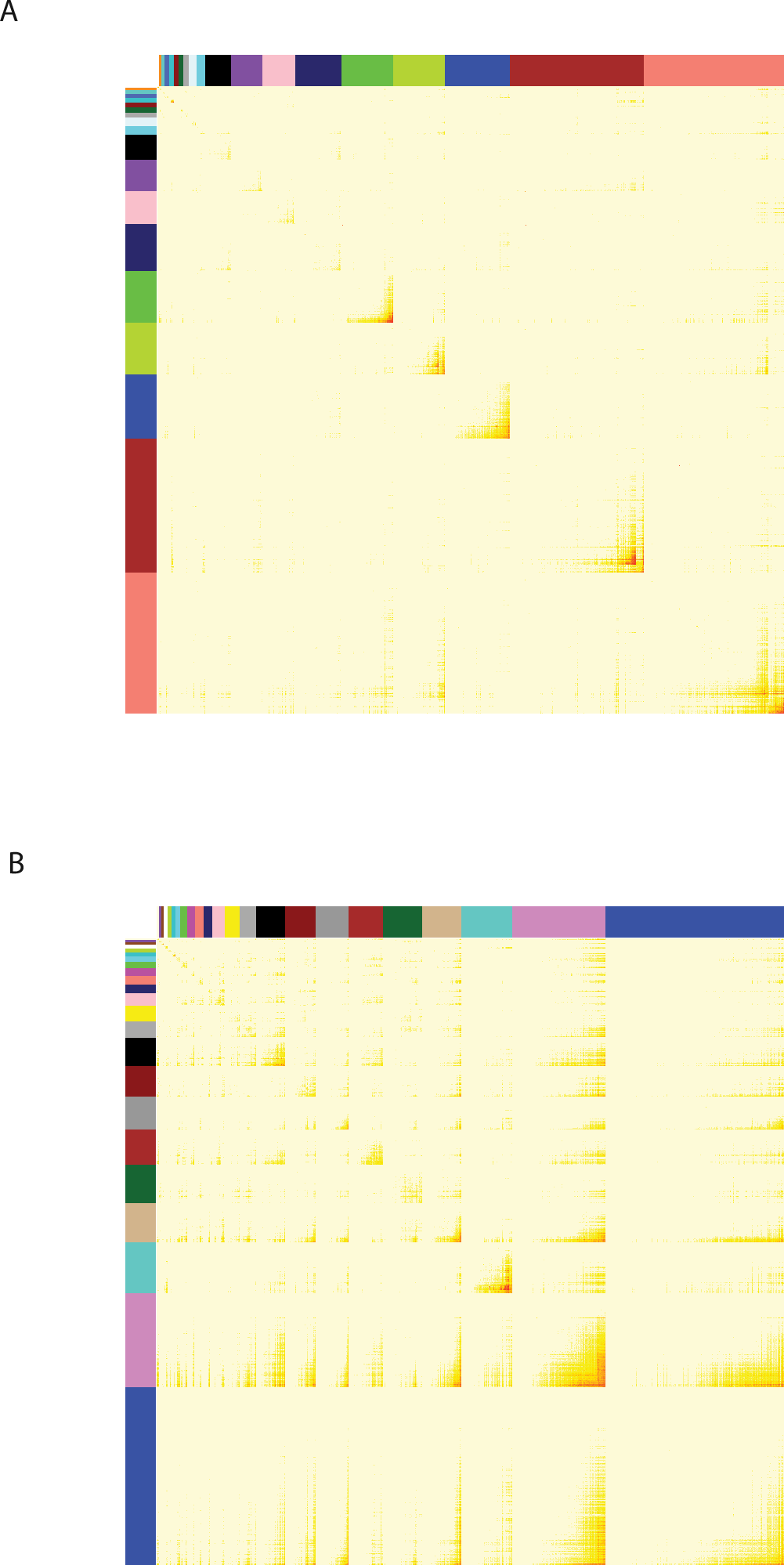

Supplement: S4 Fig — (A) Heatmap of the topological overlap between genes expressed in tumors. Each row and column represent a gene, light color indicates low topological overlap and progressively darker red indicates higher topological overlap. Module assignment is displayed along the left and the top of the heatmap. (B) Heatmap of the topological overlap between genes expressed in SR. The legend follows S4A Fig. (TIF) [file pcbi.1005680.s009.tif]

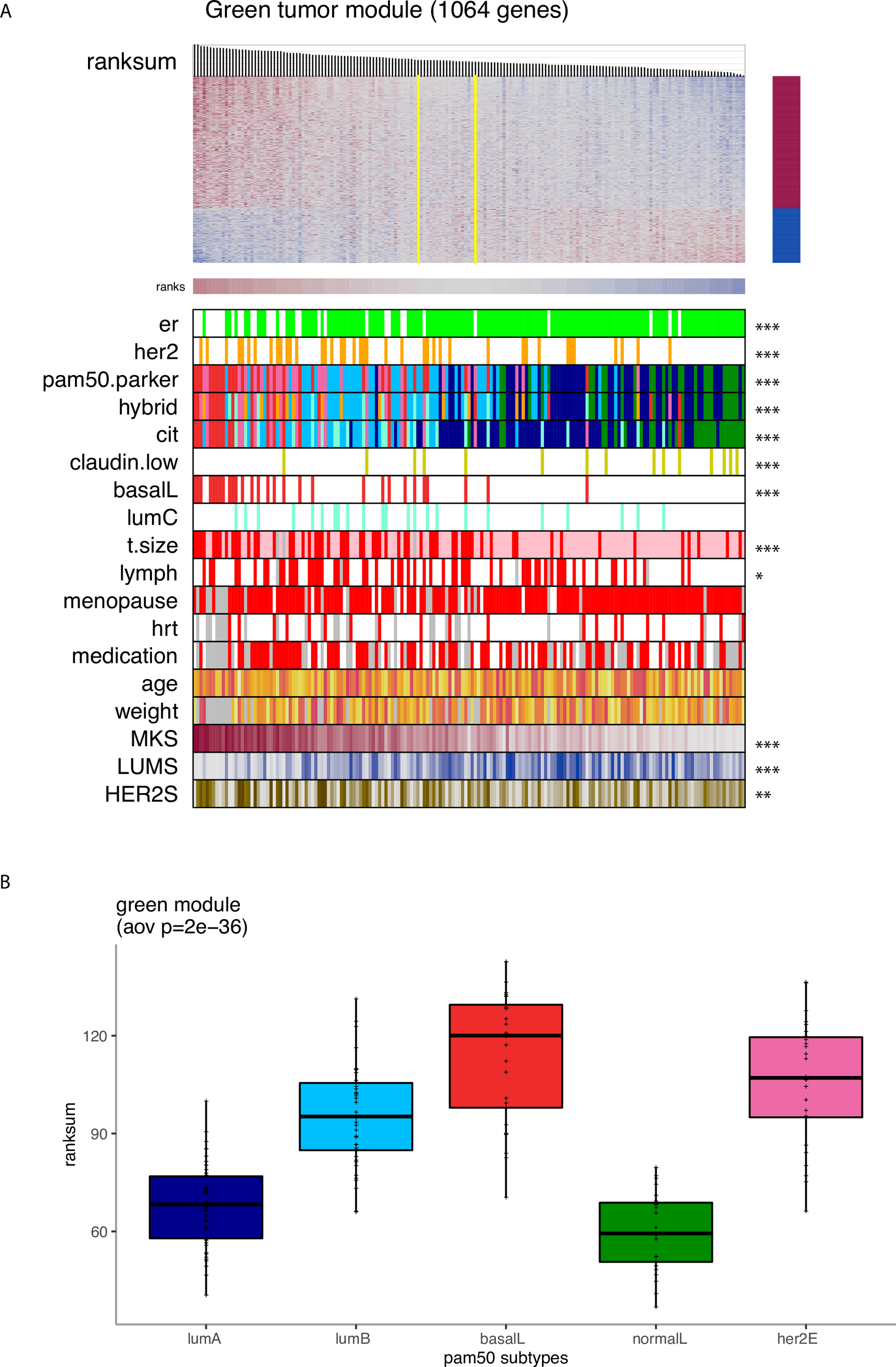

Supplement: S5 Fig — (A) Expression heatmap of genes in the green tumor module. Legend follows S3 Fig. Color coding for ER, HER2, pam50, hybrid, cit, claudin-low and lymph follows Fig 1D. In general, a tick for a binary clinical variable refers to a positive value (eg. a red tick in ‘basalL’ refers to patients with basalL tumors). For continuous variables such as Mitosis Kinase Score (MKS), Luminal Score (LUMS), HER2 score (HER2S), age, and weight, dark and light shades represent high and low values, respectively. Asterisks represent the level of significance of the associations between the gene ranksums for the green tumor module and clinicopathological attributes of patients. Associations were estimated using ANOVA or Pearson correlation for categorical and continuous variable, respectively (p-value < *0.05, **0.01, ***0.001). (B) Distribution of ranksums for the green tumor module according to pam50 subtypes. aov: analysis of variance. (TIF) [file pcbi.1005680.s010.tif]

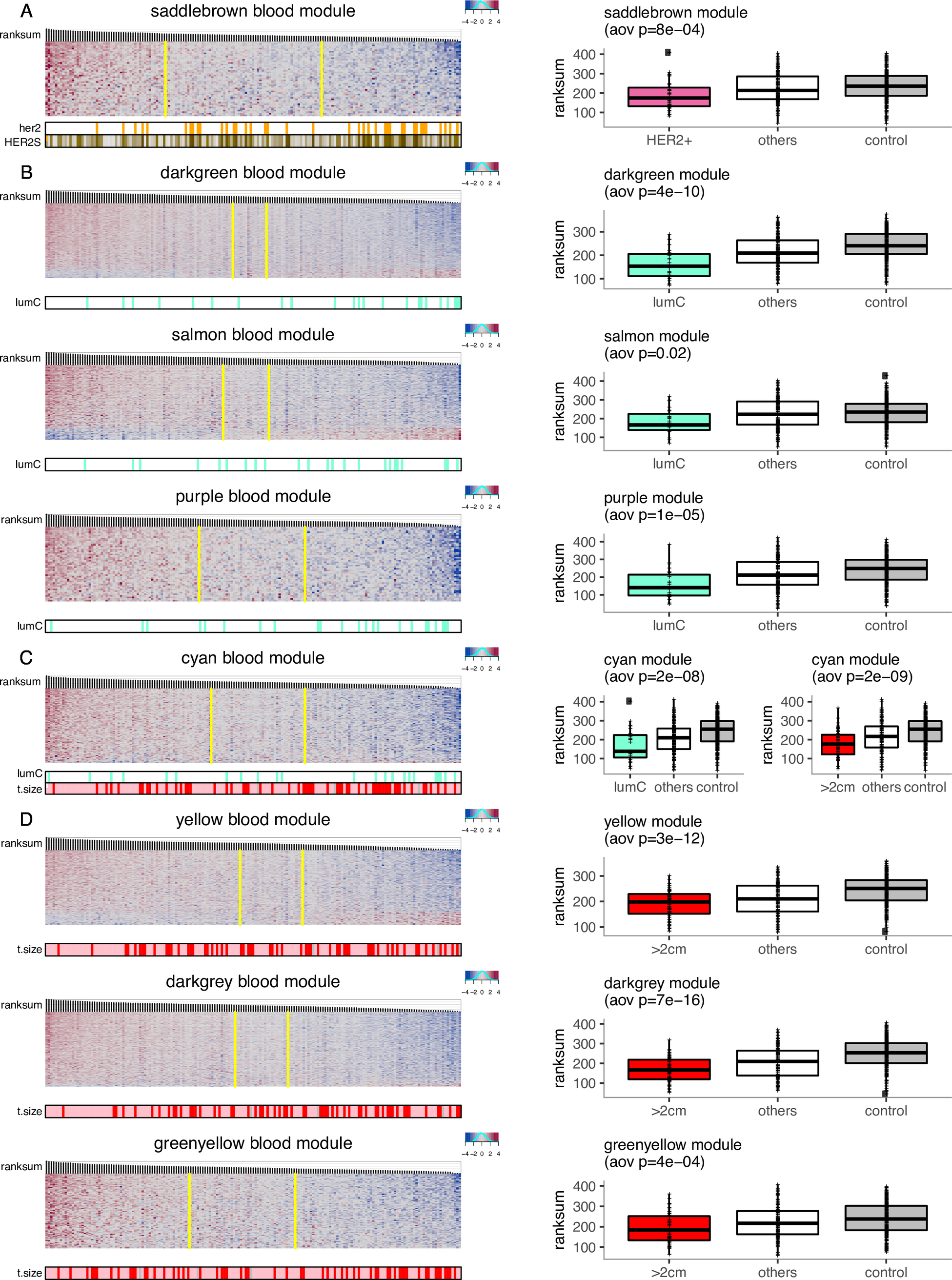

Supplement: S6 Fig — (A) One (saddlebrown) modules in the patient SR is associated with HER2+ BC. (B) Three modules in the patient SR are associated with lumC BC. (C) One module in the patient SR are associated to both lumC and large (>2cm) tumors. (D) Three modules in the patient SR are associated with large (>2cm) tumors. The legend for expression heatmaps (left) follows S3 Fig. Boxplots (right) compare module expression in SR from patients with lumC, HER2+, or large tumors with other BC patients, and controls. aov: analysis of variance. (TIF) [file pcbi.1005680.s011.tif]

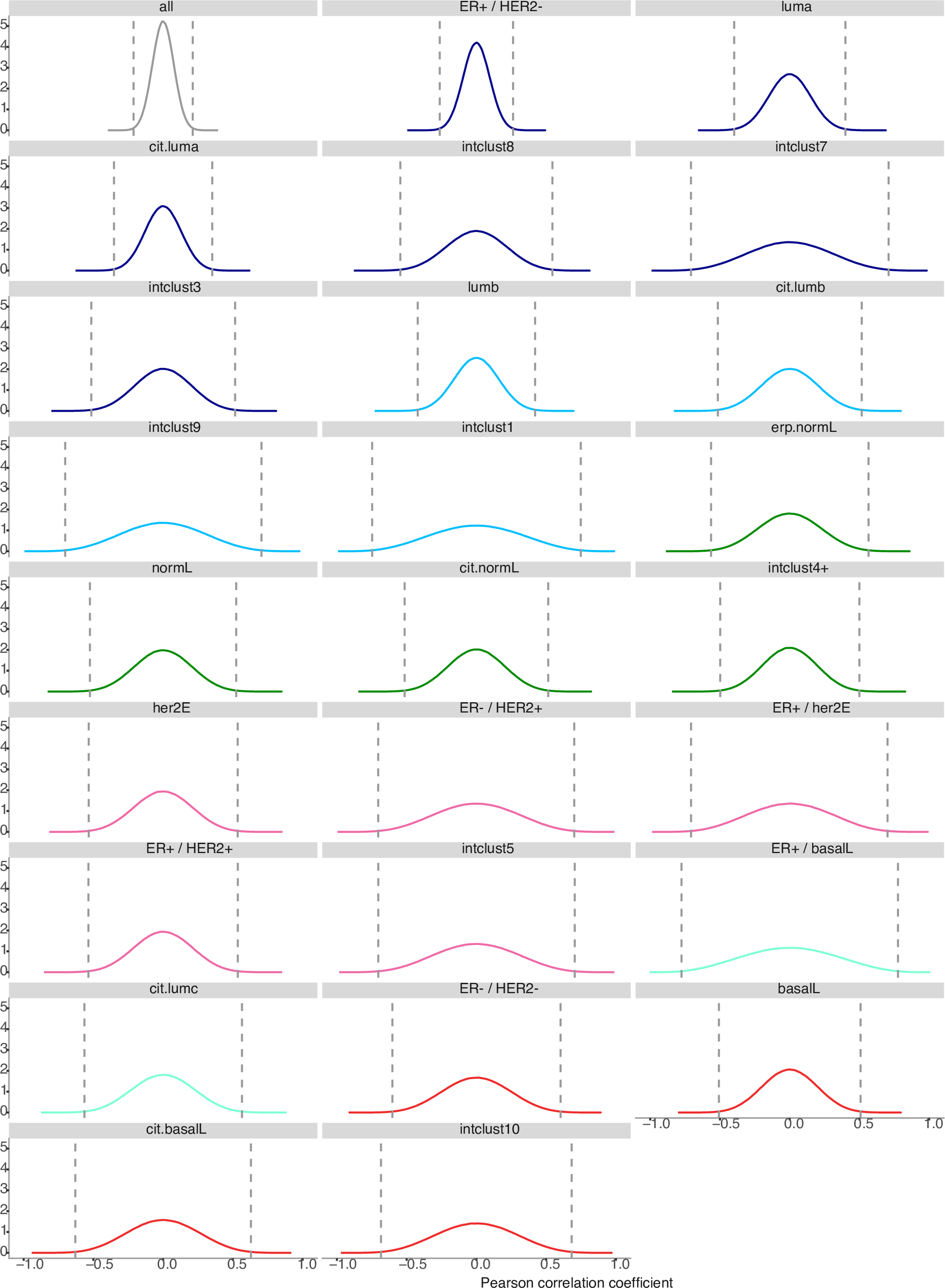

Supplement: S7 Fig — The dotted lines represent the lower and higher bounds that were used to call significant associations between modules across tissues. Curves are colored according to the families of subtypes as listed in Fig 4. (TIF) [file pcbi.1005680.s012.tif]

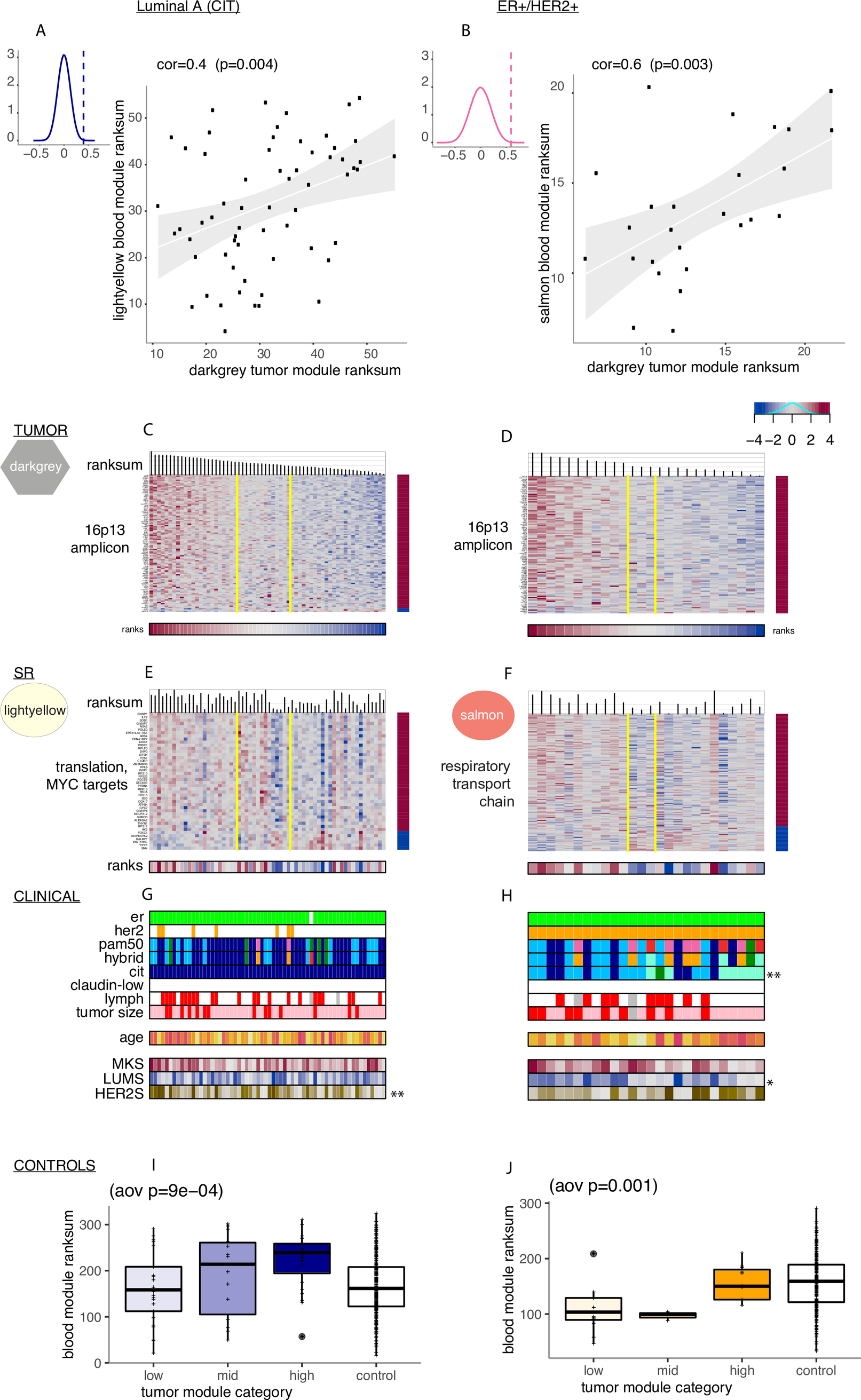

Supplement: S8 Fig — (A) Scatter plot of ranksums of the darkgrey tumor module and the lightyellow SR module in CIT lumA patients. The top corner depicts the background distributions of the correlations coefficients between ranksums of every modules pairs across tissues in CIT lumA patients. (B) Scatter plot of ranksums of the darkgrey tumor module and the salmon SR module in ER+HER2+ patients. Legend follows S8A Fig (C) Expression heatmap of genes in the darkgrey tumor module in luminal A patients under the CIT scheme [8]. Patients are linearly ordered based on the ranksum of gene expression of the darkgrey tumor module. Yellow vertical lines delimit the ROI95 in tumor that contains 95% of the randomly generated samples. Genes that are positively and negatively correlated with the patient ranksum are represented in the right sidebar colored in red and blue, respectively. Top enrichment keywords are indicated on the left of the heatmap (S2 and S3 Tables). (D) Expression heatmap of genes in the darkgrey tumor module in ER+/HER2+ patients. Legend follows S8C Fig. (E) Expression heatmap of genes in the lightyellow SR module luminal A patients under the CIT scheme [8]. Legend follows S8C Fig. Top enrichment keywords are indicated on the left of the heatmap (S4 and S5 Tables). (F) Expression heatmap of genes in the salmon SR module in ER+/HER2+ patients. Legend follows S8C Fig. Top enrichment keywords are indicated on the left of the heatmap (S4 and S5 Tables). (G) Clinical characteristics of luminal A patients under the CIT scheme [8] ordered by gene ranksums derived from the darkgrey tumor module. Legend follows Fig 1D. Asterisks represent the level of significance of the associations between the gene ranksums for the darkgrey tumor module and clinicopathological attributes of patients. Associations were estimated using ANOVA (fdr < *0.1, **0.05, ***0.01). (H) Clinical characteristics of ER+/HER2+ patients ordered by gene ranksums derived from the darkgrey tumor module. Legend follows S8G [file pcbi.1005680.s013.tif]
