# Supplementary material for: Interactions between the tumor and the blood systemic response of breast cancer patients
Source: PLoS Comput Biol. 2017 Sep 28;13(9):e1005680. doi: 10.1371/journal.pcbi.1005680 (PMC5619688; doi:10.1371/journal.pcbi.1005680)
Supplement: S1 Text — (PDF) [file pcbi.1005680.s014.pdf]

S1 Text: Supporting Methods

1. Data collection and biospecimen processing
    1.1. Sample acquisition
    1.2. Sample selection and extraction of total RNA
    1.3. Gene expression profiling and clinical data
    1.4. Microarray data preprocessing
    1.5. Clinical data
2. Gene-wise analyses to identify blood markers of subtypes
3. Weighted gene co-expression network analysis (WGCNA) and gene modules
    3.1. Co-expression networks
    3.2. Gene modules
    3.3. Functional enrichment analyses
4. Patient linear ordering and region of independence
5. Association between module expression and clinical variables
6. Association between modules
7. Data and software availability
8. Supplemental References

1. Data collection and biospecimen processing

1.1. Sample acquisition

Tumor and blood samples were obtained as part of the Norwegian Women and Cancer (NOWAC) study. NOWAC is a prospective population-based cohort that tracks 34% of all Norwegian women born between 1943-57 [49].

Between 2006 and 2010, ten of the largest Norwegian hospitals participate in collecting blood and tumor tissue from incident breast cancer (BC) cases. In collaboration with the Norwegian Breast Cancer Group, every woman born between 1943 and 1957 participating in the NOWAC study who was admitted to a collaborating hospital for a diagnostic biopsy or for surgery of BC was asked to donate, before surgery and treatment, a tumor biopsy and two blood samples, one collected into PAXgene™ tube (PreAnalytiX GmbH, Hembrechtikon, Switzerland) for gene expression analysis and another in a citrate tube. Participants were also asked to answer a two-page questionnaire eliciting information mainly on current use of hormones and medications, alcohol and smoking habits. Biological samples were then mailed overnight for biobanking at −70°C in Tromsø. In parallel, five controls were approached for each BC case in order to obtain blood samples from at least two controls per case. The controls were drawn at random but matched by time of inclusion in the NOWAC cohort and birth year. The human biological material has been approved by Regional Committees for Medical and Health Research Ethics in Norway and is in accordance with the Norwegian law on biobanking.

1.2. Sample selection and extraction of total RNA

Each year from 2008-11, blood samples were selected from the NOWAC biobank:

- In 2008, 120 blood samples from BC cases and two matched controls for each case were selected from NOWAC (bl 1).
- In 2009, 96 blood samples from cases and two matched controls for each case were selected from NOWAC (bl 2).
- In 2010, 63 blood samples received within 4 days after blood collection from cases and one matched control for each case were selected from the NOWAC (bl 3).
- In 2011, 90 blood samples received within 4 days after blood collection from cases and one matched control for each case were selected from NOWAC (bl 4).

Samples from bl 1 were sent to AROS Applied Biotechnology A/S (Aarhus, Denmark) for automated RNA extraction on the MDx Biorobot (Qiagen Inc.). Since automated extraction yielded lower RNA quantity, total RNA from subsequent blood samples (bl 2-4) were isolated using the PAXgene Blood miRNA Isolation Kit according to

the manufacturer's manual at the NTNU Genomics Core Facility in Trondheim, Norway. To control for technical variability such as different lot variations of reagents and kits, day to day variations, microarray production batches and effects related to different laboratory operators, each case was grouped with one corresponding matched control through RNA extraction (except in bl 1-2 where RNA extraction was run randomly), amplification and hybridization. Our pilot study carefully addressed the challenges of statistical methodology including intra- & inter-individual variability when working with blood gene expression profiles [35] and matched gene expression changes in blood between cases and controls included in bl 2-4 were previously investigated [33]. A careful comparison of the SR of BC patients vs their controls across the 3 latter datasets identified a gene signature that reports the presence of BC [33]. The signature is specific to BC, classifying women with other non-breast carcinoma as negative. The genes/pathways in the signature suggest how the SR may be involved in the progression of BC, eg cytostatic immune-related signal in BC patients.

In 2011, 327 biopsy samples were selected from the NOWAC biobank and total RNA/DNA were extracted using the AllPrep DNA/RNA mini kit (Qiagen Inc, Hilden, Germany) following the the manufacturer's manual at the Norwegian Radium Hospital in Norway (t 1).

1.3. Gene expression profiling and clinical data

Gene expression profiling of blood and tumor samples was performed at the NTNU Genomics Core Facility in Trondheim, Norway. RNA quantity and purity was assessed using the NanoDrop ND-8000 spectrophotometer (ThermoFisher Scientific, Wilmington, Delaware, USA) and Agilent bioanalyzer (Palo Alto, CA, USA), respectively. RNA amplification was performed in 96 plates using 300 ng of total RNA and the Illumina® TotalPrep™-96 RNA Amplification Kit (Ambion Inc., Austin, TX, USA). Cases and one matched control per case included in blood data 2 (n = 190) and 3 (n=126) were profiled on the IlluminaHumanAWG-6 version 3 expression bead chips. Cases and one matched control per case included in bl 1 (n = 160) and 4 (n=180) as well as tumor samples (n=306) were profiled on the IlluminaHumanHT-12 version 4 expression bead chips. GenomeStudio from Illumina (San Diego, CA, USA) was used to assess the quality of each array.

Clinical data from cases, all histological type confounded, from which we have a biopsy were provided by the cancer registry (as of Dec, 31 2014). The clinical elements include: estrogen and human epidermal growth factor receptors (ER, HER2), histological type (eg ICD-0 85003 for invasive ductal carcinoma), tumor size, stage and lymph node status. Receptor statuses were curated from pathological report for 21 patients included at the University Hospital of North Norway. Curated information was in agreement with information provided by the cancer registry and allowed us to assign 5 cases as HER2 negative (HER2 -) that had missing cancer registry information.

1.4. Microarray data preprocessing

Microarray data preprocessing and analysis were performed using R v3.2.0 (http://cran.r-project.org), RStudio (http://www.rstudio.com/), and tools from the Bioconductor project (http://www.bioconductor.org) [68], adapted to our needs.

Preprocessing of the microarray data was performed identically in each dataset separately using the summary data from GenomeStudio and the limma package as described in Richie et al. [69]. The datasets were trimmed of samples found misdiagnosed after update from the cancer registry and NOWAC database, samples with extremely low proportion of probes expressed above the level of the negative controls, or found outliers before and after normalization (S1A Fig). More precisely, outliers were identified when their euclidean distance to the cluster center was larger than twice the median distances to the center. The cluster center was defined by the average of all samples after removing 10% samples farthest away from the center. We then applied the normal-exponential convolution model [70] to calibrate the background level, normalize and transform the probe intensities from each sample within each dataset.

The Illumina annotation packages by Barbosa-Morais [71] provide access to a reannotation pipelining based on the 50-base sequence and a scoring system that quantifies the reliability of each probe. Using the Illumina arrays version 3 (for bl 2 and 3), and version 4 (for bl 1,4 and t 1), we removed probes assigned as "Bad" or "No match" quality score and the most highly expressed probe among probes with identical Entrez Gene ID was selected (S1A Fig).

Automated RNA extraction for samples in blood data 1 was done on two plates that created a technical batch effect that we corrected using the limma package [69]. The function in effect fits a linear model to the data including batches and then removes the component due to the batch effects. Before adjustment, we confirmed that study groups (case/control, pam50 subtypes) were evenly distributed across (plates) batches. We then translated probe id into gene names and selected genes in common across all blood and tumor datasets.

We then selected BC cases to be invasive ductal carcinoma and from which we have both blood and tumor profiles (S1A Fig). To have enough patients in each BC subtype, the four blood datasets including BC patients were merged

into one dataset. We also constructed another dataset merging the four blood datasets including BC patients and controls.

To adjust for batch effect caused by blood dataset of origin, gene intensities were adjusted for batch effect using the Combat method [72]. To avoid over-correcting blood data for BC patients and deflate results from our main analyses (comparing BC subtypes), we included pam50 subtypes as a covariate in our model after making sure that subtypes were evenly distributed across (dataset) batches (chisq test p=0.7) [73]. A more conservative approach was used in blood data from BC patients and controls where no covariate was used to adjust data, although study group was also here evenly distributed across datasets (chisq.test p=0.8).

In total, we investigated blood and tumor profiles from 172 BC patients and blood profiles from 290 controls. Profiles include 16,782 unique genes.

1.5. Clinical data

Among the 265 BC cases of any histopathological type with good quality tumor profiles, ~20% and ~60% had missing ER and HER2 status information from the cancer registry, respectively. To impute missing values, we used the expression of the *ESR1* gene and the expression of genes included in the HER2 amplicon (*ERBB2, GRB7, PGAP3, PNMT, MIEN1, TCAP*) in tumor samples to rank patients, and after which we constructed a receiver operating characteristic (ROC) curve setting using IHC/FISH assignment as true label. We then selected the threshold which resulted in false positive rate < 0.2 with regard to the true label (S1B-C Fig) [74].bianchiniThe proliferation score was calculated as the average expression of 12 mitotic kinases to produce the Mitotic kinase gene expression score (MKS) as previously described [45]. The luminal score (LUMS) and the HER2 score (HER2S) were similarly calculated using pam50 luminal genes (*MAPT, FOXA1, ESR1, PGR, BAG1, NAT1, MLPH)* and the above-mentioned genes members of the HER2 amplicon.

Tumor subtypes were constructed for invasive ductal carcinoma cases only from which we have a good quality tumor profiles (n= 208). All patients were assigned an intrinsic subtype, Luminal A (abbreviated here as lumA), Luminal B (lumB), Normal-like (normalL), Basal-like (basalL), and HER2-enriched (her2E) via the PAM50 method [5]. togihWe also partitioned patients into seven hybrid subtypes [9]. For ER+ tumors, intrinsic subtyping is used for further refinement: ER+/lumA, ER+/lumB, ER+/normalL, ER+/her2E, and ER+/basalL. For ER-tumors, HER2 status is used: ER-/HER2+ and ER-/HER2-. CIT [8] assignments were obtained using the "citbcmst" R/Bioconductor package where probes were mapped via HGNC symbols. The IntClust subtyping scheme [6] is based on gene expression and high density DNA copy number profiles simultaneously but we inferred intclust subtypes using RNA-based surrogate algorithm [7,39]. Claudin-low assignments were computed as described previously [75]. Briefly, each dataset was merged with the cell line dataset of Prat et al. using the Distance Weighted Discrimination (DWD) software [76] prsavailable at https://genome.unc.edu/pubsup/dwd/. Next, claudin-low was assigned to samples using the DWD function SSP→correlation with euclidean distance and the claudin-low centroids available at https://genome.unc.edu/pubsup/clow/CLAUDIN-LOW-PREDICTOR/.

2. Gene-wise analyses to identify blood markers of subtypes

The R package Limma was used to obtain lists of differentially expressed genes in blood between patients in a single target tumor marker / subtype / metasubtype versus all patients without that marker / subtype / metasubtype. In this manner, we investigated

- tumor clinical markers: ER+ *vs* ER- , HER2+ *vs* HER2-
- single pam50 subtype: lumA *vs* others, lumB *vs* others, normalL *vs* others, her2E *vs* others, basalL *vs* others
- single hybrid subtype: ER-/HER2- *vs* others, ER-/HER2+ *vs* others, ER+/lumA *vs* others. ER+/lumB *vs* others, ER+/normalL *vs* others, ER+/her2E *vs* others, Er+/basalL *vs* others.
- single CIT subtypes: lumA *vs* others, lumB *vs* others, normalL *vs* others, lumC *vs* others,mApo vs others, her2E *vs* others, basalL *vs* others.
- proliferative metasubtype: (lumB∪BasalL∪her2E) *vs* (lumA∪normalL)
- luminal metasubtype: (lumA∪lumB) *vs* (normalL∪her2E-basalL)
- luminal-normal metasubtype: (lumA∪lumB∪normalL) *vs* (her2E∪basalL)
- CIT basalL-mApo metasubtype: (cit.basalL∪cit.mApo) *vs* (cit.lumA∪cit.lumB∪cit.lumC∪cit.normalL)
- profilerative CIT luminal metasubtypes: (cit.lumB∪cit.lumC) *vs*

(cit.lumA∪cit.normalL∪cit.basalL∪cit.mApo)

- Single IntClust subtype (n patients >10): IntClust1 *vs* others, IntClust3 *vs* others, Intclust4+ *vs* others, IntClust5 *vs* others, IntClust7 *vs* others, Intclust8 *vs* others, Intclust9 *vs* others, IntClust10 *vs* others.

For each test, p-values were adjusted for multiple testing using false discovery rate [65].

3. Weighted gene co-expression network analysis (WGCNA) and gene modules

3.1. Co-expression networks

Since network analysis and module detection can be biased by outlier gene expression values, we set to missing each expression value $X_{(i,j)}$ for gene i and patient j if :

$$| X_{(i,j)} - median\,(X(i)) | > k \times MAD\,(Xi),$$

where *MAD* is the median absolute deviation and k is a constant threshold. k was set to 4.65 so 99% of all expression values in tumors, where MAD are larger than in blood, remain in our analysis.

All pairwise correlations were then calculated for genes in blood and breast tumor tissue, respectively, and converted into measures of connection strength by taking their absolute values and raising them to a power, β [41]. To make meaningful comparisons across data sets, a power of β = 6 (default value) was chosen for both tumor and blood. We selected β such that an approximate scale-free topology [77] is achieved (model fit index $R^2 \geq 0.8$) in both networks.

3.2. Gene modules

To identify modules of coexpressed genes, we searched for genes with similar patterns of connection strengths to other genes or high ‘topological overlap’ (TO) [42]. We calculated (signed) TO and clustered genes on this basis for both blood and breast tumor tissue. The tree was cut to define modules (min size > 30 genes) using the the Dynamic Hybrid Tree Cut algorithm [43] (height = 0.99 and deepSplit = 2). This resulted in 41 and 27 modules in blood and tumor, respectively (S3 Fig). Approximately the same number of genes in both tissue were not assigned to any module and therefore excluded (n = 3,882 and 3,863 genes in blood and tumor, respectively). Modules were further merged if the correlation between their 1st principal components was > 0. 75.

For visual analysis of the constructed networks and gene modules, the nodes and edges with TO > 0.1 were represented using ggnet2 R package using the fruchterman-reingold layout and Cytoscape 3.2.1 [47] using the edge-weighted spring embedded layout (2A-B Fig).

3.3. Functional enrichment analyses

Modules in each network were characterized using several strategies. First, modules were annotated based on gene ontology (GO) enrichment using the R topGO package [44]. Enrichment was evaluated by the ‘weight01’ algorithm that assesses, prune and weigh for local dependencies of GO terms, and Fisher exact test. The main principle of the weight01 algorithm is to reinforce differences in significance between a node *u* and its neighbors. S2, S4 Tables report the first 5 most significant nodes using the ‘weight01’ algorithm and Fisher exact test for each module in each tissue.

Second, modules were further annotated based on MSigDB signatures (v5.1) in the hallmark (h), curated (c2), oncogenic (c6) and immunological (c7) gene set collections [66]. We used additional genes sets of interest including peripheral-blood mononuclear cell (PBMC) transcriptional modules [52], our blood-based gene expression signatures (341- and 50-gene) for BC [33], and immune-specific gene sets from [46]. Enrichment for each gene signature was estimated for all genes in the modules and for genes that are positively (red genes up) or negatively (blue genes dn) correlated to the patient ranksum only using the hypergeometric minimum-likelihood P-values, computed with the function ‘dhyper’ (equivalent to one-sided Fisher exact test). P-values were then adjusted for multiple testing using false discovery rate [65] (S3, S5 Tables). All results with p-value < 1 are available at http://mixt-blood-tumor.bci.mcgill.ca.

4. Patient linear ordering and region of independence

Our approach is to map *n* samples to a linear ordering based on expression of *k* genes within a given module:

1. Genes are partitioned into two groups around myeloids (*M1*and *M2*) using correlation as the distance metric.
2. Each gene in *M1* and *M2* is ordered from high to low and low to high expression, respectively.
3. Expression values of each gene are then replaced by *(n,...,1)*rank.
4. The sum of gene ranks (ranksum) are then used to linearly ordered patients.

For module *M*, we can distinguish three categories of samples:

1. Samples whose expression of genes in *M1*are greater than average, while the reverse is true for the expression of genes in *M2*. These samples have high ranksum values.
2. Samples whose expression of genes in *M* are independent and do not exhibit the correlation structure indicated by the partition of *M* into *M1*and *M2*.
3. Samples whose expression of genes in *M1* are lower than average, while the reverse is true for the expression of genes in *M2*. These samples have low ranksum values.

We propose the following random sampling procedure for partitioning the samples into the three above-mentioned (high, mid, low) categories given *k* genes in a module *M* :

1. We extend the rank matrix with a new 'artificial' patient 'n + 1'
2. Each of the *k* genes rank patient 'n+1' with a uniformly randomly chosen number from *(1,.... ,n+1)*.
3. Summing the randomized rank over all k genes in *M*, the position of patient 'n+1' is computed within the observed linear order. This is repeated a suitably large number of times (n = 10,000). The region of independence (ROI_{95}) is determined by the 0.025 and 0.975 percentile point of the distribution of random patient ranks.

For each module, a 'heatmap' of gene expressions is produced with rows corresponding to genes and columns corresponding to samples (linearly ordered as described above). To determine the gene ordering in the heatmap, we calculate how each gene expression correlates with the observed patient ordering. Red genes at the top and blue genes at the bottom of the heatmap have expressions that correlates the most positively and negatively with the patient ordering, respectively.

5. Association between module expression and clinical variables

Using ranksums to capture module expression, we asked how modules in each tissue are differentially expressed according to patient's clinicopathological variables. The type of the clinicopathological attribute (categorical or continuous) determines the underlying statistical test. Pearson correlation (Student asymptotic p-value) was used to test association between between a given module and continuous patient attributes (eg. age, weight, MKS, LUMS). Analysis of Variance (ANOVA) was used to test association between a given module and categorical patient attributes (eg. ER, HER2, subtypes, LN). For each variable. we computed empirical p-values by permuting clinical labels (n=1000).

For each variable, we perform 42 association tests (23 blood modules + 19 tumor modules). We use false discovery rate [65] to correct for multiple testing for each variable independently or for each "family" of tests. We define families of tests when the dependent variables are very similar as follow:

- family 1: tests for pam50, cit, hybrid as these RNA-based subtyping schemes are highly similar (S1 Table).
- family 2: tests for ER and LUMS since it codes for about the same characteristics but the variable is categorical and continuous, respectively.
- family 3: tests for HER2 and HER2S since it codes for about the same characteristics but the variable is categorical and continuous, respectively.

The other variables (lymph node status, lumC, tumor size, claudin-low, hormone replacement therapy (hrt), menopause, medication use, age, weight, and MKS) were considered independently.

6. Association between modules

Fisher exact test was used to test the significance of gene overlap between modules. P-values were adjusted for multiple testing using the false discovery rate [65].

Pearson correlation (permutation-based p-value) between patient ranksums was used to test association between modules. To determine association between modules within a given subtype, we first stratify on subtypes and then rerun the MIxT procedure: (i) computation of ranksums in each module, (ii) calculation of the correlation between ranksums and (iii) in each module, 10K permutation of ranksums to estimate significance of correlation between modules.

ANOVA was used to compare blood module expression between BC patients (assigned to a defined tumor module ROI categories) and controls.

7. Data and software availability

Gene expression data have been deposited at the European Genome-phenome Archive [67] (EGA; https://www.ebi.ac.uk/ega/ ; accession number EGAS00001001804).

The MIxT web application (http://mixt-blood-tumor.bci.mcgill.ca/) is written in the Go programming language to provide an interface to statistical analyses in R and link to online databases. Users can browse through all the results generated for this study, visualize gene co-expression networks and expression heatmaps, and search for genes, gene lists, and pathways. We use Bootstrap (http://getbootstrap.com) to build the user interface and Javascript libraries D3 (http://d3js.org) and Sigma (http://sigmajs.org) to build interactive visualizations. The web application framework is open sourced at http://github.com/fjukstad/mixt.

8. Supplemental References

References1-67 in main manuscript

68. Huber W, Carey VJ, Gentleman R, Anders S, Carlson M, Carvalho BS, et al. Orchestrating high-throughput genomic analysis with Bioconductor. Nat Methods. 2015;12(2):115-21. doi: 10.1038/nmeth.3252. PubMed PMID: 25633503; PubMed Central PMCID: PMCPMC4509590.
69. Ritchie ME, Dunning MJ, Smith ML, Shi W, Lynch AG. BeadArray expression analysis using bioconductor. PLoS computational biology. 2011;7(12):e1002276. doi: 10.1371/journal.pcbi.1002276. PubMed PMID: 22144879; PubMed Central PMCID: PMCPMC3228778.
70. Shi W, Oshlack A, Smyth GK. Optimizing the noise versus bias trade-off for Illumina whole genome expression BeadChips. Nucleic acids research. 2010;38(22):e204. doi: 10.1093/nar/gkq871. PubMed PMID: 20929874; PubMed Central PMCID: PMCPMC3001098.
71. Barbosa-Morais NL, Dunning MJ, Samarajiwa SA, Darot JF, Ritchie ME, Lynch AG, et al. A re-annotation pipeline for Illumina BeadArrays: improving the interpretation of gene expression data. Nucleic acids research. 2010;38(3):e17. Epub 2009/11/20. doi: 10.1093/nar/gkp942. PubMed PMID: 19923232; PubMed Central PMCID: PMC2817484.
72. Johnson WE, Li C, Rabinovic A. Adjusting batch effects in microarray expression data using empirical Bayes methods. Biostatistics. 2007;8(1):118-27. doi: 10.1093/biostatistics/kxj037. PubMed PMID: 16632515.
73. Nygaard V, Rodland EA, Hovig E. Methods that remove batch effects while retaining group differences may lead to exaggerated confidence in downstream analyses. Biostatistics. 2016;17(1):29-39. doi: 10.1093/biostatistics/kxv027. PubMed PMID: 26272994; PubMed Central PMCID: PMCPMC4679072.
74. Sing T, Sander O, Beerenwinkel N, Lengauer T. ROCR: visualizing classifier performance in R. Bioinformatics. 2005;21(20):3940-1. doi: 10.1093/bioinformatics/bti623. PubMed PMID: 16096348.
75. Prat A, Parker JS, Karginova O, Fan C, Livasy C, Herschkowitz JI, et al. Phenotypic and molecular characterization of the claudin-low intrinsic subtype of breast cancer. Breast cancer research : BCR. 2010;12(5):R68. Epub 2010/09/04. doi: 10.1186/bcr2635. PubMed PMID: 20813035; PubMed Central PMCID: PMC3096954.
76. Benito M, Parker J, Du Q, Wu J, Xiang D, Perou CM, et al. Adjustment of systematic microarray data biases. Bioinformatics. 2004;20(1):105-14. PubMed PMID: 14693816.
77. Albert R. Scale-free networks in cell biology. J Cell Sci. 2005;118(Pt 21):4947-57. doi: 10.1242/jcs.02714. PubMed PMID: 16254242.
